# Precise control over gas-transporting channels in zeolitic imidazolate framework glasses

**DOI:** 10.1038/s41563-023-01738-3

**Published:** 2023-12-20

**Authors:** Oksana Smirnova, Seungtaik Hwang, Roman Sajzew, Lingcong Ge, Aaron Reupert, Vahid Nozari, Samira Savani, Christian Chmelik, Michael R. Reithofer, Lothar Wondraczek, Jörg Kärger, Alexander Knebel

**Affiliations:** 1 University of Jena, Otto Schott Institute of Materials Research, Jena, Germany; 2 University of Leipzig, Faculty of Physics and Earth Sciences, Leipzig, Germany; 3 University of Vienna, Institute of Inorganic Chemistry, Faculty of Chemistry, Institute of Inorganic Chemistry, Vienna, Austria; 4 Center of Energy and Environmental Chemistry—CEEC Jena, University of Jena, Jena, Germany

**Keywords:** Metal-organic frameworks, Chemical engineering, Glasses, Metal-organic frameworks

## Abstract

Porous metal–organic frameworks have emerged to resolve important challenges of our modern society, such as CO_2_ sequestration. Zeolitic imidazolate frameworks (ZIFs) can undergo a glass transition to form ZIF glasses; they combine the liquid handling of classical glasses with the tremendous potential for gas separation applications of ZIFs. Using millimetre-sized ZIF-62 single crystals and centimetre-sized ZIF-62 glass, we demonstrate the scalability and processability of our materials. Further, following the evolution of gas penetration into ZIF crystals and ZIF glasses by infrared microimaging techniques, we determine the diffusion coefficients and changes to the pore architecture on the ångström scale. The evolution of the material on melting and processing is observed in situ on different length scales by using a microscope-coupled heating stage and analysed microstructurally by transmission electron microscopy. Pore collapse during glass processing is further tracked by changes in the volume and density of the glasses. Mass spectrometry was utilized to investigate the crystal-to-glass transition and thermal-processing ability. The controllable tuning of the pore diameter in ZIF glass may enable liquid-processable ZIF glass membranes for challenging gas separations.

## Abstract (web summary)

Porosity of zeolitic imidazolate frameworks can be preserved beyond glass transition and melt processing. Here centimetre-scale porous glasses are demonstrated, whereas liquid processing enables fine-tuning of the size of the gas-transporting channels for molecular sieving.

## Main

As the industrial interest in porous metal–organic frameworks (MOFs) rises, the combination of their complex properties with those of other material classes is shifting paradigms of both worlds. Controlled burning of MOFs offers highly potent catalysts^1^ and colloid chemistry yields liquids with permanently accessible porosity inside a solvent^2,3^. Another highly promising material originates from a subclass of MOFs called zeolitic imidazolate frameworks (ZIFs), which were found to be meltable into glasses. This nucleated the field of hybrid MOF glasses less than 10 years ago^4,5^. Glasses have triggered a range of societal revolutions over the past 30,000 years and now, hybrid glasses from MOFs or other types of coordination compound^6^ have been identified as the next potential breakthrough in energy and environmental technologies^7^. It was proposed that ZIF glasses combine the porous features of MOFs and the universal processability of classical glasses in their liquid state^2^. However, the residual pore architecture in ZIF glass is not yet understood, and the glass-like melt processing of MOF objects or products has not been demonstrated to date. Although a range of properties typical for glassy materials (such as mechanical characteristics) were tested on MOF glass specimens^8,9^, the fundamental question of porosity in these glasses remains unresolved.

Crystalline ZIFs are already outstanding molecular sieves and are very well suited for gas separation in membranes: only their soft porous features lead to drawbacks^2,10^. Here we show that ZIF glasses indeed lay the foundation for the perspective applications of glasses in gas separation, as they offer tailorable molecular-sieving properties with a higher degree of precision than reticular materials themselves^11^. The possible disruptive impact of an amorphous molecular-sieving material with highly defined size exclusion is immense^12^, as it allows to tackle all kinds of separation task, from methane valorization and carbon capture^13^ to chemical separation technologies operating at the limits of size-exclusion sieving^10^.

The glass transition of ZIFs was originally discovered on ZIF-4. It was proposed and later confirmed through X-ray scattering experiments that the associated melt-quenched ZIF glasses exhibit free-space cavities^14^. Since then, other ZIFs and tetrahedral imidazolate frameworks (TIFs), such as TIF-4, ZIF-62, ZIF-76 and ZIF-8 have been found to be meltable. A logical approach towards porous glasses has been taken with ZIF-8 and ZIF-76, which show sodalite cages with high porosity^15,16^. An imidazole-based ionic liquid helped not only to transform unmeltable ZIF-8 into a glass and to decrease the melting temperature of ZIF-76 (ref. ^17^) but also led to non-accessible porosity from the decomposed guest inside the glass^18^. For ZIF-62—probably the best-known MOF glass former to date—the currently available data do not give a clear insight into the porous microstructures in glass. ^67^Zn nuclear magnetic resonance (NMR) seems to prove that there is no short-range order in ZIF-62 glass and thus no leftover porosity in re-melted glasses to resemble its MOF origin^19^. On the other hand, conflicting conclusions were drawn from X-ray scattering for glasses directly derived from the crystalline state^20^. Nevertheless, the adsorption of permanent gases and hydrocarbons and pores as big as 9 Å were reported for ZIF-62 glasses^21^. ZIF-62 has been synthesized on ceramic supports, and the glasses made from it achieve pore cut-offs at about 3.3 Å (refs. ^22,23^); similarly, ZIF-4/TIF-4 were applied in gas separation membranes^24^.

The route to ZIF-62, and from a crystal to a_g_ZIF-62 (a_g_, amorphous glass) differs from scientist to scientist. Often described are problems in phase purity, where ZIF-zni phases occur during synthesis^25^. Even the smallest impurities result in strong variations in material properties, for example, in thermal stability or optical appearance^8,26^. Notable variations in thermal processing methods, such as melt-quenching rates, re-melting of glass or direct collapse of MOF could be the reason why some groups report non-porous glasses, whereas others predict MOF-like features^27^. However, currently reported MOF glasses suffer from serious material quality issues, including bubble formation, (micro-)cracking, surface oxidation and partial decomposition^28^.

To us, the biggest misconception in terms of porosity determinations in ZIF glasses is the frequently applied volumetric gas adsorption to characterize porosity. MOFs (such as ZIF-62) with already limited surface areas and pore volumes, demonstrate even 50% reduction in porosity as glass^29^, rendering them uninteresting for adsorptive gas separation. However, for membrane separation, the plain molecular-sieving properties are of the highest interest, whereas pore volume and surface area are only secondary parameters. Our goal was to investigate the pore channels instead, the number of pathways a gas can take and how these are altered on liquid processing.

Measuring kinetic gas uptake as gas diffusion tracked in situ through infrared microscopy (IRM) in single crystals has shown to be particularly feasible for mixed-linker MOFs^30^ such as ZIF-62. It is also feasible to fully characterize glasses^31^ and the gas transport properties in nanoporous materials following Fick’s laws of diffusion^32^. Based on our findings, we can now rationally understand the evolution of the gas-transporting pore channels on the processing of ZIF glasses, and even find strong evidence that the microstructure, and thus the gas transport properties, can be altered in a controlled way until a total size exclusion for a large gas species is achieved.

## Scalable synthesis of porous ZIF-62 and ZIF-62 glasses

**Fig. 1:**
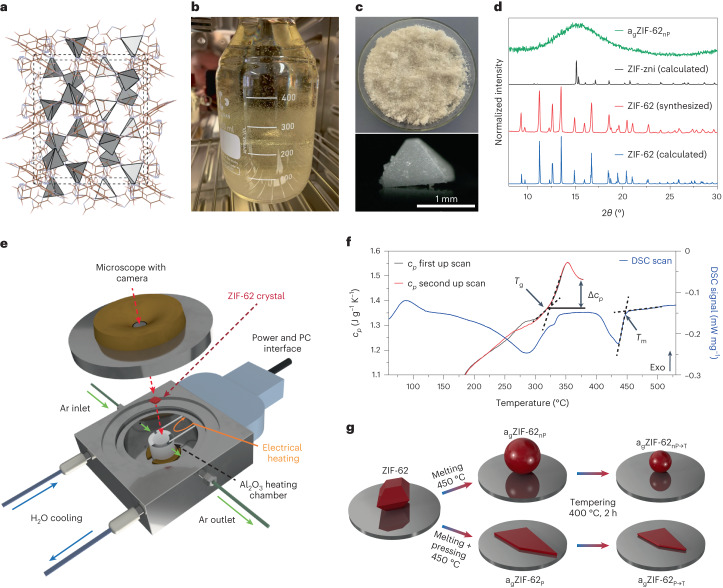
Synthesis, manufacturing and processing of ZIF-62 and derived glasses. **a**, Crystal structure of ZIF-62 in the *a* direction. **b**, Photograph of upscaled ZIF-62 synthesis with large crystals growing on the walls. **c**, Photograph of as-synthesized 10 g ZIF-62(Zn) from a single-batch synthesis and micrograph of a typical crystal. **d**, PXRD data of ZIF-62 and a_g_ZIF-62_nP_ and simulated ZIF-zni and ZIF-62. **e**, Schematic of the in situ heating stage for the optical microscope. **f**, DSC signal to determine *T*_m_ of the ZIF-62 batch and cyclic scans of heat capacity *c*_*p*_ with heating and cooling rates of 20 °C min^−1^ to determine *T*_g_. **g**, Flow chart for melt processing applied to ZIF-62 in this study. ZIF-62 and the derived materials are shown in red. Source data

Previously reported ZIF-62(Zn) synthesis (Fig. 1a shows the structure) is commonly performed in small batches^28,29^ or suffers from severe phase impurities, mainly the ZIF-zni phase^25^.

Our upscaled synthesis procedure (Fig. 1b,c and Supplementary Information) results in 10 g (15 g in total after reusing the mother liquor twice) of phase-pure ZIF-62(Zn) with crystals up to 2 mm in size (Fig. 1c and Supplementary Figs. 1 and 2). Instead of preparing the stock solutions as it has been frequently reported^20,27,28,33^, a direct preparation was used. We chose the highest-purity-grade chemicals and added the two ligands one by one, followed by adding the Zn^2+^ precursor to the well homogenized mixture (Supplementary Information). Inhomogeneous mixing with imidazole (Im)-rich regions would lead to zni-phase crystallization^25^. The linker ratio of the as-synthesized ZIF-62 was calculated from NMR^21^ to contain the ratio of benzimidazole (bIm) to imidazole (Im) of 1.0:4.8 (Supplementary Fig. 3) and the formation of ZIF-62 was confirmed using powder X-ray diffraction (PXRD)^34^ (Fig. 1d). The synthetical scaling up is also possible for ZIF-62(Co) (Supplementary Figs. 4 to 29), but the material is not further investigated in this report. Washing and drying procedures are of key importance for obtaining high-quality ZIF-62 materials with only 0.02 dimethylformamide (DMF) molecules per unit cell as determined by ^1^H NMR (Supplementary Fig. 5). This amount of DMF seems to be structure directing for the crystal matrix^35^. Differential scanning calorimetry (DSC) and heat capacity (*c*_*p*_) measurements show the melting point (*T*_m_) and glass transition temperature (*T*_g_) (Fig. 1f). As our ZIF-62 has higher purity, we find a 10–30 K increase in *T*_m_ = 450 °C and *T*_g_ = 322 °C compared with previous reports^4,16,20,21,25,29,35^.

Melting was investigated in situ with a microscope-coupled heating stage performed under an Ar atmosphere using single crystals (Fig. 1e); a flow chart (Fig. 1g) describes our procedures. Properly washed and vacuum-dried ZIF-62 then formed transparent and nanoporous glasses on melting (Methods). We think that problems, such as bubble formation^20,28^ and even exploding crystals (Supplementary Video 1), may occur because of the decomposition of excess residual DMF that acts as a blowing agent. Transparent a_g_ZIF-62_nP_ (nP, not pressed) is obtained through direct crystal melting (Supplementary Videos 2 and 3), whereas flat and homogeneously transparent glass shards of a_g_ZIF-62_P_ (P, pressed) are obtained by applying a mechanical load during the melting process (Supplementary Figs. 10 and 11). Both a_g_ZIF-62_nP_ and a_g_ZIF-62_P_ were then processed by tempering at 400 °C, further noted as a_g_ZIF-62_nP→T_ (Supplementary Video 4) and a_g_ZIF-62_P→T_ (Supplementary Video 5), respectively (Fig. 1g). Ex situ optical micrographs were always taken before and after the processes (Supplementary Figs. 12–16).

## Kinetic gas uptake in processed a_g_ZIF-62

**Fig. 2:**
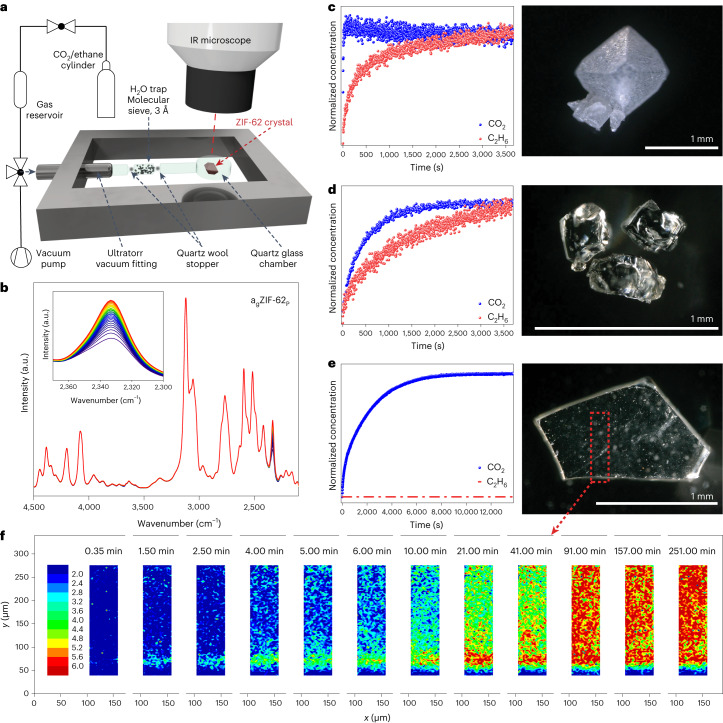
Investigation of kinetic gas diffusion by in situ IRM. **a**, Schematic of the in situ IRM setup. **b**, Typical IR absorbance spectra to determine the kinetic gas uptake, visualizing the increase in the CO_2_ peak over time (for a_g_ZIF-62_P_). **c**–**e**, Normalized kinetic gas uptake curves for 0–40 mbar CO_2_ and 0–200 mbar ethane in ZIF-62 single crystal (**c**), not-pressed a_g_ZIF-62_nP_ (**d**) and pressed a_g_ZIF-62_P_ (**e**), with their corresponding microscopy images. **f**, Guest concentration mapping on CO_2_ uptake over 250 min in a_g_ZIF-62_P_ on the edge of a glass shard (blue colour means relatively low CO_2_ concentration and the gradient to red colour with high CO_2_ concentration). Source data

Kinetic gas uptake was measured for 0–40 mbar CO_2_ and 0–200 mbar ethane, and their diffusion into the sample matrix was followed by IRM (Fig. 2a,b and Supplementary Figs. 17–21)^31,32^. Each series of measurements was performed on a single crystal or a single glass piece, which avoids any influence from grain boundary diffusion.

The accessible porosity in the neat ZIF-62 crystal allows for very fast CO_2_ uptake with a kinetic diameter of 3.3 Å. Moreover, ethane (4.16 Å) can enter the pores, too (Fig. 2c). In this case, a kinetic difference is visible, which is attributed to the different sizes of the molecules. Our data show that the pore channels in crystalline ZIF-62 are much better accessible than previously thought^22,34,36^. As for the amorphous samples, we still find a relatively fast uptake in a_g_ZIF-62_nP_ for CO_2_, and slower uptake for ethane (Fig. 2d), which demonstrates selectivity. This again is contradictory to prior findings, where the pore diameters, and therefore the apertures, get substantially larger in a_g_ZIF-62 (refs. ^21,22,37^), but also to other references stating that pores in a_g_ZIF-62 are inaccessible^19,38^.

The CO_2_ diffusivity in a_g_ZIF-62 is two orders of magnitude slower compared with neat ZIF-62, because of a steady shrinking of the pore channels. When melt pressing a_g_ZIF-62_P_, the CO_2_ uptake becomes even slower than for a_g_ZIF-62_nP_. No ethane uptake was detected in a_g_ZIF-62_P_ and total exclusion was achieved. We show that compressing the liquid ZIF-62 glass results in smaller pore channels, which completely stops ethane from entering (Fig. 2e). These findings show a sharp molecular-sieving cut-off between CO_2_ and ethane with tremendous potential for molecular sieving.

Gas diffusion into a glass shard of a_g_ZIF-62_P_ (thickness, 200 µm) was investigated by guest concentration mapping through infrared (IR) microimaging^39^, monitoring the evolution of a CO_2_ distribution inside the sample (Fig. 2f). In the separate IR maps, the local CO_2_ concentration is tracked by IR signals of guest molecules in the glass shard at different times from 0.35 to 251.00 min. This dataset confirms that the change in pore channels in a_g_ZIF-62_P_ is macroscopically homogeneous throughout the sample. Moreover, this experiment reveals the propagation of CO_2_ molecules not only through the top and bottom surfaces but also through the side of the glass shard (parallel to the long axis).

For both CO_2_ and ethane, a clear trend is observed: diffusion rate decreases with processing ZIF-62 > a_g_ZIF-62_nP_ > a_g_ZIF-62_P_. These results prove that the pore architecture of ZIFs is strongly dependent on and is even controllable by the liquid-handling process.

## Microstructural investigation of porosity channels

**Fig. 3:**
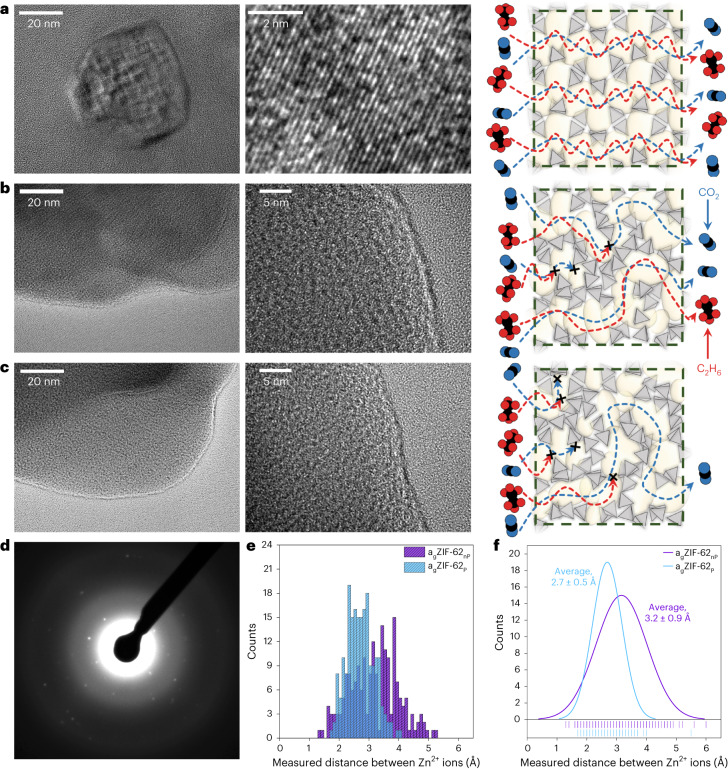
Microstructural investigation of the porosity channels by TEM. **a**–**c**, HR-TEM images with two different magnifications and schematic of the presumable changes in the porous structure and gas pathways in ZIF-62 (**a**), a_g_ZIF-62_nP_ (**b**) and a_g_ZIF-62_P_ (**c**). In the schematic, the blue molecules represent CO_2_ and the red molecules, ethane. **d**, ED pattern of crystalline ZIF-62 from **a**. The ‘x’ marks in the schematics in **a**–**c** visualize completely blocked diffusion pathways. **e**, Histogram of 200 distance measurements between two mass centres (by contrast; bright spot, atomic position of Zn^2+^), giving the interatomic distances from the HR-TEM data in **b** and **c**. **f**, Normal distribution of the histogram in **e**, giving an average value for the interatomic distance of 3.2 Å in a_g_ZIF-62_nP_ and 2.7 Å in a_g_ZIF-62_P_. Extended Data Figs. 1 and 2 show a comparison of the TEM analysis for the data in **e** and **f**. Source data

**Extended Data Fig. 1:**
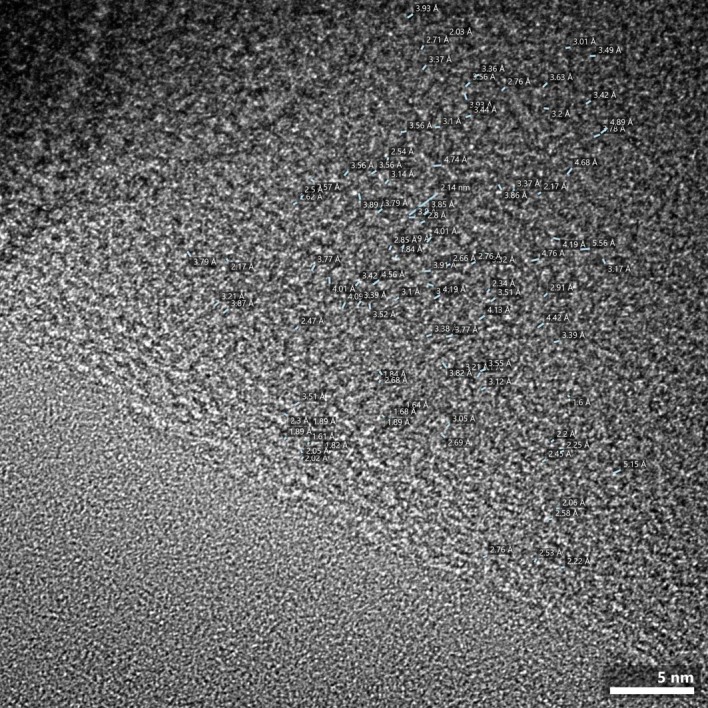
Interatomic distances in a_g_ZIF-62_nP._ The statistical values of the interatomic distances in a_g_ZIF-62_nP_ obtained through HR-TEM with blue lines corresponding to the distance measured.

**Extended Data Fig. 2:**
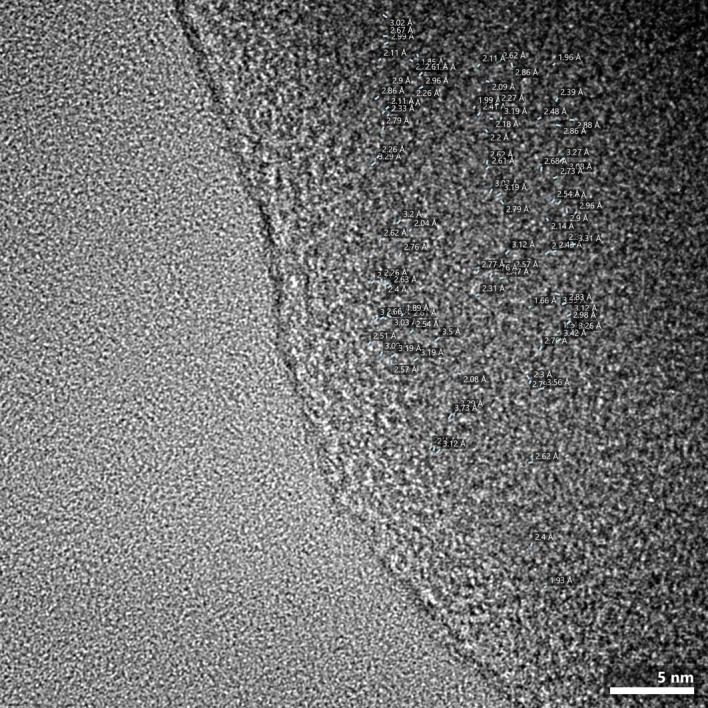
Interatomic distances in a_g_ZIF-62_P._ The statistical values of the interatomic distances in a_g_ZIF-62_P_ obtained through HR-TEM with blue lines corresponding to the distance measured.

High-resolution transmission electron microscopy (HR-TEM) images of ZIF-62 (Fig. 3a), a_g_ZIF-62_nP_ (Fig. 3b) and a_g_ZIF-62_P_ (Fig. 3c) were obtained to investigate the microstructural evolution throughout the glass processing. For ZIF-62, we find lattice planes and collect the crystal electron diffraction (ED) pattern of a single crystal (Fig. 3d). Supplementary Figs. 22 and 23 show the halo-like ED patterns of both amorphous samples. On melting, the ordered structure of the crystal is lost, but pore channels remain, which are visible in the microstructure in the HR-TEM data of a_g_ZIF-62_nP_ (Fig. 3b). The HR-TEM data for a_g_ZIF-62_P_ (Fig. 3d) at the same scale demonstrate a higher density of these microstructures. The interatomic distances were measured over clearly distinguishable void spaces and therefore can be assumed as an indicator for the average pore-limiting diameter in the glass samples (Extended Data Figs. 1 and 2). The measured interatomic distances show the decrease in the pore-limiting diameter from ~3.5 Å (ref. ^21^) for crystalline ZIF-62 to 3.2 Å for a_g_ZIF-62_nP_ and 2.7 Å for a_g_ZIF-62_P_ (Fig. 3e,f).

Supported by the results from the HR-TEM microstructural analysis and gas diffusion (Fig. 2), we propose a pore channel evolution and gas diffusion mechanism next to each sample (Fig. 3a–c).

## Tempering of ZIF glass

**Fig. 4:**
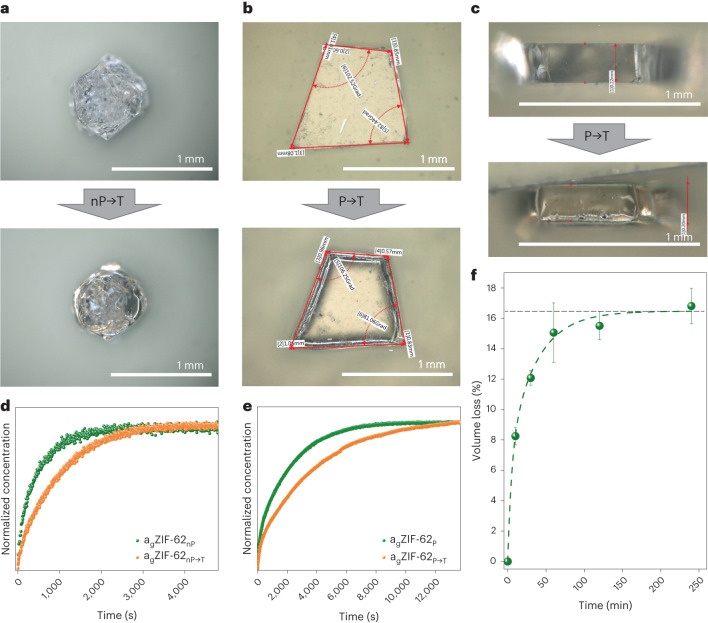
Influence of tempering on porosity in a_g_ZIF-62. **a**, Micrographs of the same a_g_ZIF-62_nP_ sample before and after tempering a_g_ZIF-62_nP→T_. **b**, Top-view microscopy images of the a_g_ZIF-62_P_ sample before and after tempering a_g_ZIF-62_P→T_. **c**, Micrographs of the side views corresponding to the data in **b**. **d**, Kinetic CO_2_ uptake from 0 to 40 mbar measured by the IRM for a_g_ZIF-62_nP→T_ and a_g_ZIF-62_nP_. **e**, CO_2_ uptake from 0 to 40 mbar measured by the IRM for a_g_ZIF-62_P→T_ and a_g_ZIF-62_P_. **f**, Volume loss over tempering time for geometrically measured (ex situ) a_g_ZIF-62_P→T_ samples, with the size of a_g_ZIF-62_P_ taken for point (0,0). The error bars displayed here are non-sampling and are observation errors estimated from the error propagation within all the individual parameters and measurements. The horizontal dashed line indicates the maximum volume loss found during tempering from the exponential decay fitting function. Source data

To determine the influence of temperature and time in the liquid state on the pore channels, we heat-treated previously obtained a_g_ZIF-62_nP_ and a_g_ZIF-62_P_ to tempered a_g_ZIF-62_nP→T_ and a_g_ZIF-62_P→T_ above *T*_g_ at *T* = 400 °C. Tempering affects both types of sample in a similar way, resulting in smoothened edges due to surface tension of the melt, but also in some visible shrinking because of pore collapse. However, both a_g_ZIF-62_nP→T_ and a_g_ZIF-62_P→T_ remain transparent after tempering (Supplementary Fig. 24 shows the ultraviolet–visible transmittance spectra), with the appearance of only a slight yellow shade (Fig. 4a–c).

CO_2_ uptake and diffusion on the tempered samples was measured by IRM. As expected, a stronger pore collapse leads to slower CO_2_ diffusion (Fig. 4d,e). In both a_g_ZIF-62_nP→T_ and a_g_ZIF-62_P→T_, tempering leads to a stronger partial collapse of the pores, making an increasing number of channels and pores inaccessible for the gas molecules. However, we still find CO_2_ diffusing through both samples; moreover, a_g_ZIF-62_nP→T_ (Supplementary Fig. 25) still adsorbs ethane, whereas the pore structure of a_g_ZIF-62_P→T_ remains completely inaccessible for ethane. Guest concentration mapping on CO_2_ uptake into a_g_ZIF-62_P→T_ visually demonstrates that the diffusion occurs slower, compared with agZIF-62_P_, and less amount of CO_2_ is needed to reach the saturation level (Fig. 2f and Supplementary Fig. 26).

Five shards of the same a_g_ZIF-62_P_ sample were observed in the tempering process of a_g_ZIF-62_P→T_ for different times up to 4 h (Methods and Supplementary Information). Tempering resulted in the shrinking of the a_g_ZIF-62_P→T_ sample in all three dimensions (Supplementary Figs. 12–16 and Supplementary Table 1). These experiments show a rapid volume loss of about 16% with increasing tempering time that reaches a plateau after 2 h. An exponential decay function was used to fit the decay of the volume (Fig. 4f). Further, the shrinking is optically visible on micrographs after 4 h, showing surfaces comparable with cooled-down lava (Supplementary Fig. 16 and Supplementary Video 5). When handling a_g_ZIF-62_nP_ in an air atmosphere, all the gas diffusion rates increase as a partial decomposition of glasses occurs (Supplementary Figs. 27 and 28), which could explain the contrary findings^19,21^.

This demonstrates that a controlled tempering program leads to the tailoring of molecular-size-dependent transport in ZIF-62 glass. Through variations in temperature, duration and mechanical load, ZIF-62 can potentially be controllably altered towards ‘universal molecular sieving’ for gases that do not chemically damage the material.

## Diffusivities in ZIF-62 and a_g_ZIF-62 materials

Diffusivities were calculated for five samples, namely, ZIF-62, a_g_ZIF-62_nP_, a_g_ZIF-62_P,_ a_g_ZIF-62_nP→T_ (2 h) and a_g_ZIF-62_P→T_ (2 h), based on kinetic CO_2_ and ethane uptake (wherever applicable) (Table 1). Although ethane uptake was clearly observed in a_g_ZIF-62_nP_ and a_g_ZIF-62_nP→T_ (Supplementary Fig. 25), determining its diffusivity was not flawlessly possible due to the extremely slow uptake—it did not reach equilibrium within the given experiment duration of 6 h, possibly related to capillary condensation, a fairly common effect in porous materials^40^. As expected, crystalline ZIF-62 shows the highest diffusivity, which differs from all other samples by at least two orders of magnitude; the lowest belongs to a_g_ZIF-62_P→T_. Among a_g_ZIF-62_nP_, a_g_ZIF-62_P_ and a_g_ZIF-62_nP→T_, CO_2_ diffusivities do not differ noticeably and are more pronounced for a_g_ZIF-62_P→T_ (Table 1).

**Table 1:** Measured physical properties of ZIF-62, a_g_ZIF-62_nP_, a_g_ZIF-62_P_, a_g_ZIF-62_nP→T_ and a_g_ZIF-62_P→T_

Physical property	ZIF-62	a_g_ZIF-62_nP_	a_g_ZIF-62_P_	a_g_ZIF-62_nP→T_	a_g_ZIF-62_P→T_
Diffusivity, CO_2_ (m^2^ s^−1^)	5.12 × 10^−10^	3.53 × 10^−^^12^	2.81 × 10^−12^	2.09 × 10^−12^	8.92 × 10^−13^
Diffusivity, ethane (m^2^ s^−1^)	3.82 × 10^−12^	n.a.*	n.a.	n.a.*	n.a.
Skeletal density (g cm^−3^)	1.4728	1.4163	1.5250	1.4248	1.5316
	1.3903	← Corrected by thermogravimetric mass loss (5.6 wt%)			
Envelope density (g cm^−3^)	1.4178	1.3469	1.4458	1.3557	1.4556
	1.3384	← Corrected by thermogravimetric mass loss (5.6 wt%)			

Diffusion coefficients of CO_2_ and ethane (*uptake measured, but diffusivity is impossible to calculate due to extremely slow uptake) and skeletal and envelope densities.

## Nature of ZIF-62 crystal-to-glass transition

We used helium (He) pycnometry to determine the skeletal densities of crystals and glasses including He-accessible porosity. Further, we measured the envelope density of the samples following Archimedes’ principle using bulky toluene, which cannot penetrate into the material^2^, to include the porosity of the matrix.

**Fig. 5:**
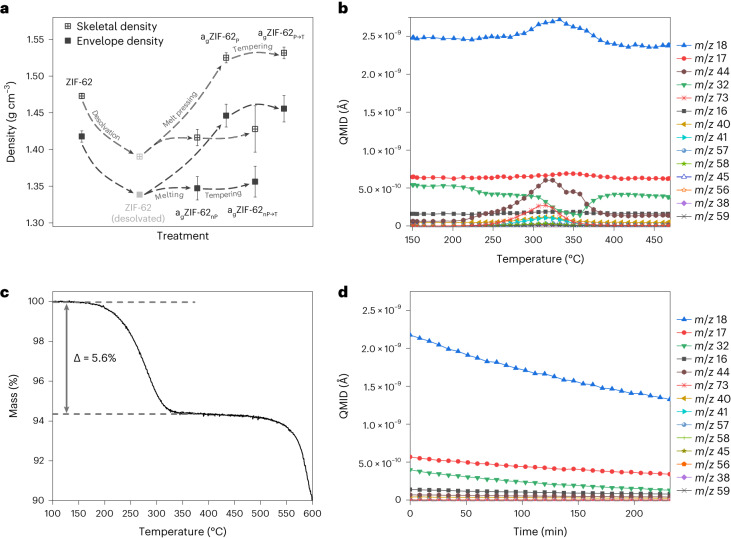
Crystal-to-glass transition in ZIF-62. **a**, Skeletal and envelope densities of ZIF-62 and ZIF-62-derived samples following the treatment indicated on the *x* axis. The error bars displayed here are non-sampling and are observation errors estimated from the error propagation within all the individual parameters and measurements. **b**, MS data for ZIF-62 on heating above the melting point. QMID, quasi-multiple-ion detection; *m*/*z*, mass divided by charge number. **c**, TGA/DSC mass-loss curve for ZIF-62. **d**, MS data for a_g_ZIF-62_nP_ tempering. Source data

As expected, the skeletal He density in porous materials is always higher than the envelope density^41^. In case of ZIF-62 and ZIF-62 glasses, the densities change proportionally (Fig. 5a). All the density values are listed in Table 1. The envelope density of a_g_ZIF-62_nP_ of 1.3469 g cm^−3^ is in good accordance with recently discovered data, namely, 1.3500 g cm^−3^, as determined from CO_2_ sorption experiments^37^. The envelope and skeletal densities of crystalline ZIF-62 were found to be 1.4178 and 1.4728 g cm^−3^, respectively, being noticeably higher than the calculated crystallographic density from the same work (1.2900 g cm^−3^) (ref. ^37^). We suppose that such a contradiction might be caused by the difference between the simulated ‘empty’ crystalline matrix and the real object. Based on the thermogravimetric analysis (TGA)/DSC data (Figs. 1f and 5c), a 5.6% mass loss occurs at the thermal amorphization point of ZIF-62 at 330 °C. We think that amorphization before the glass transition^35^ is based on the desolvation of structurally important guests. Similar to ZIF-90 (ref. ^42^), the ZIF-62 structure seems to be supported by guest molecules with limited periodicity. Therefore, the mass loss at the initial amorphization point needs to be considered crucial for the destabilization of ZIF-62 and the values must be corrected for a guest-free crystal density (Fig. 5a). We investigated the mass loss during melting by DSC-coupled mass spectrometry (MS), which shows that it is only associated with the desolvation of guest molecules. Therefore, low amounts of DMF and its fragments leave the crystal, but also—unexpectedly, as ZIF-62 should be hydrophobic—water is confirmed by the MS data (Fig. 5b). A burst release of water molecules and CO_2_ from the framework is found from 320 to 330 °C, meaning that these molecules are strongly adsorbed in the framework. Interestingly, the base level of oxygen shows a sudden loss later at 350 °C after the amorphization temperature, which might be attributed to capping the open metal sides in the amorphized framework. After that point, melting at 450 °C happens without any mass loss. Additionally, we measured the DSC-MS data for tempering at 400 °C for a_g_ZIF-62_nP_ (Fig. 5d) and a_g_ZIF-62_P_ (Supplementary Fig. 29) over 240 min. Neither desolvation nor decomposition with mass loss occurs during tempering. Overall, we detect neither any linkers disappearing from the lattice nor any decomposition of the remaining structure under inert conditions below 500 °C. These facts show that the liquid handling of a_g_ZIF-62 and longer-lasting temperature treatments are destruction free and processing is possible.

## Discussion

The ZIF glass would have strong benefits over a polycrystalline ZIF-62 film: no more grain boundaries that lead to defect diffusion, and very good molecular-sieving capabilities even though ZIF glass is an amorphous material. Through the demonstrated scalability of bulk ZIF-62, liquid glass processing paves the way for large-scale productions. By changing the processing parameters of ZIF-62 glass, the collapse of the pores is highly controllable and allows to reach an ångström-scale resolution. This may become extremely interesting when looking at challenging separations, such as H_2_/CH_4_ or He/CH_4_, in the future. Further interesting applications are all kinds of CO_2_ separation, such as H_2_/CO_2_, N_2_/CO_2_ or even direct CO_2_ capture from air, which demand highly precise molecular sieving. With control over the microporous channels in ZIF glasses, we might be able to develop a liquid-processable, free-standing ZIF glass membranes made from 100% bulk ZIFs and tackle modern, important and highly challenging gas separations.

## Methods

### Synthesis of ZIF-62 (Zn and Co)

The synthesis of both Zn and Co versions of ZIF-62 was based on previously reported procedures^28,29^, but some important improvements were applied. To synthesize the Zn version, 12.79 g benzimidazole was dissolved in 480 ml DMF, followed by adding 38.02 g imidazole. The mixture was stirred for 5 min and then 19.93 g Zn was added to the solution, which was stirred until complete dissolution. The resulting synthetic Zn:Im:bIm molar ratio had an approximate value of 3:25:5. The solution was transferred into a 500 ml glass jar with a DMF-resistant lid and placed in the furnace for 60 h at 130 °C. After cooling down, the white sediment was separated from the solvent using centrifugation (10 min, 8,528*g*) and thoroughly washed two times with DMF and two times with dichloromethane. The product was then transferred to a Petri dish and placed on the heating plate (60 min, 45 °C) to evaporate the extra dichloromethane. The final step was to place ZIF-62 into the vacuum furnace (25 mbar, 150 °C, 72 h) for activation. The yield was 9.86 g. By reusing the mother liquor twice, we were able to get an additional 3.92 and 0.91 g after 7 days of synthesis, thereby increasing the total yield to almost 15.00 g.

The synthesis of ZIF-62(Co), as well as washing and drying procedures, was the same as for the Zn version, but with different amounts of reagents: 6.34 g cobalt(ii) nitrate hexahydrate, 1.04 g benzimidazole and 4.15 g imidazole for 480 ml DMF. The resulting synthetic Zn:Im:bIm molar ratio was approximately 5:14:2. The procedure yielded 3.6 g.

### ZIF-62 glass manufacturing and processing

Sphere-like particles of a_g_ZIF-62_nP_ were obtained using the Linkam TS1500 instrument with a T95-HT-controller-based heating stage attached to the microscope. Up to 50 mg of the as-synthesized ZIF-62 crystals was put into the 0.06 ml platinum crucible, which was placed into the heating chamber. The lid of the heating stage was properly sealed, and Ar and water supply (as the cooling agent) were established. ZIF-62 was heated up to 450 °C at a rate of 50 °C min^−1^ and then held at this temperature for 5 min and cooled down at the same rate.

To produce the pressure-processed samples of a_g_ZIF-62_P_, some amount of gently ground ZIF-62 was placed between two silica cover glasses, which were fixed together by two metal clamps. The melting was performed in the tube furnace in the atmosphere of nitrogen. The samples were slowly heated up to 450 °C and held at this temperature for 5 min.

Samples were tempered under an Ar atmosphere using the heating stage. For each experiment, the samples were heated up to 400 °C at a rate of 50 °C min^−1^ and then held at this temperature for a determined time (from 10 to 240 min) and cooled down at the same rate. To collect the videos, Zeiss AXIO Imager Z1m with a ×5 or ×10 Epiplan-Neofluar objective was focused on the sample through the lid. An image was automatically taken every 10–30 s. A video of the heating, holding and cooling processes could then be assembled from the individual images.

### Characterization

Optical micrographs of the samples were captured using the digital microscope VHX-6000 by Keyence with a universal zoom lens VH-Z100UR (magnification range from ×100 to ×1,000) and VHX-S650 free-angle observation system (*XYZ* motorized) as a stand. The PXRD data were obtained using a Rigaku MiniFlex diffractometer with a 600 W X-ray generator providing Cu Kα radiation with a wavelength of 1.54059 Å. The Bragg–Brentano geometry in the 5.00–50.00° 2*θ* range with a step size of 0.02° was used.

The ultraviolet–visible transmittance spectra of a_g_ZIF-62_P_ and a_g_ZIF-62_P→T_ tempered for 4 h were collected using a double-beam spectrophotometer (Cary 5000, Agilent). The free optical path through air was used as the reference beam. Both samples were polished before the measurements.

The IRM instrument (Hyperion 3000, Bruker Optik) connected to a vacuum Fourier transform IR spectrometer (VERTEX 80v, Bruker Optik) with a polychromatic IR source was used to collect the IR spectra. The microscope is equipped with two separate detectors: one is a conventional single-element mercury cadmium telluride detector, which is applied to obtain the IR absorbance spectra or conduct time-resolved measurements by adjusting the size and position of a rectangular aperture on the area of interest, and the other is a focal plane array detector, which is used for IR imaging. This detector has an array of 128 × 128 single detectors with a size of 40 µm × 40 µm each. By magnifying the power of the scanning objectives to ×15, a spatial resolution of ~2.7 µm × 2.7 µm is obtained.

Bruker Avance III with a BBFO probe 400 MHz spectrometer was used to obtain the ^1^H NMR spectra. A stock solution of DMSO-d_6_ (3.000 ml) and DCl (20%)/D_2_O (0.889 ml) was prepared. Then, 6 mg of each sample (ZIF-62(Zn) and ZIF-62(Co)) was dissolved in 0.7 ml of the stock solution via 3–5 min sonication. The spectra were calibrated using TMS as a reference, and all data processing was performed using the MestReNova x64 software version 14.3.0. The linker ratio and DMF amount determination was performed similar to a previously reported procedure^21^.

### Thermal analyses

TGA, DSC and cyclic *c*_*p*_ measurements were performed using a Netzsch STA 449 F1 thermal analyser. Aluminium or platinum crucibles were used to heat the samples to the target temperature in an inert nitrogen atmosphere (flow, 20 ml min^−1^) with a heating rate of 10–20 °C min^−1^. To perform cyclic *c*_*p*_ measurements, a baseline and sapphire reference scan were collected before the sample scan using the same temperature program. The sample was first melt-quenched from 460 °C in the same crucible after collecting the sapphire scan. Afterwards, the cyclic runs were performed by heating the glass up to 380 °C and then cooling to 180 °C, and subsequent heating to 380 °C. *T*_g_ is defined as the onset temperature of the glass transition feature.

Quadrupole mass spectrometer QMS 403 Aёolos by Netzsch coupled with TGA-DSC Netzsch STA 449 F1 was used to detect masses in the *m*/*z* range of 0–120, with an acquisition rate of 40 spectra per second.

### Diffusion measurements

The sample was placed in an IR optical cell (Infrasil, Starna; cell inner diameter, 19 mm; cell height, 5 mm). The cell was mounted on the motorized sample stage under the focus of the IR microscope, and it was connected to a static vacuum system (stainless steel, one-fourth of an inch and Swagelok tubing and valves) consisting of a pump (HiCube 80 Classic, Pfeiffer Vacuum), a cylindrical gas reservoir filled with guest molecules and pressure transducers that measure the pressure inside the cell in the range from 10^−6^ to 10^4^ mbar.

For the activation of the sample, before each IR measurement, the sample was heated up to 120 °C at a heating rate of 1 °C min^−1^ under a vacuum and maintained for at least 15 h. Then, the sample was slowly cooled down to 30 °C.

After the activation of the sample, a chosen molecule—that is, either CO_2_ or ethane—was collected in the cylindrical gas reservoir at a desired pressure. The uptake was commenced by opening the entrance valve placed between the gas reservoir and IR optical cell. The pressure change within the IR optical cell was achieved within a fraction of a second after opening the entrance valve, and the pressure was effectively maintained at the desired value until the end of the measurement due to the relatively large volume of the gas reservoir compared with that of the IR optical cell.

For time-resolved measurements, the single-element mercury cadmium telluride detector was selected and different numbers of loops and scans were used depending on the speed of the uptake. For example, for CO_2_ uptake in a_g_ZIF-62_P_, 2,000 loops and 48 scans were used, meaning that there were 2,000 data points to plot the uptake curve and each data point was an average value of 48 scans. Absorbance is directly proportional to concentration according to Beer–Lambert law:

$$A=\varepsilon {cd},$$

where *A* is the absorbance, *ε* is the molar absorption coefficient of the guest species and *d* is the optical path length. Therefore, by integrating the characteristic peak area of the guest molecule under the IR absorbance spectra, the uptake curve, that is, concentration (in arbitrary units) versus time, was obtained. Then, it was normalized with the final value of the concentration, which was in equilibrium with the external pressure, to obtain a ‘normalized’ uptake curve.

### IR microimaging

a_g_ZIF-62_P_ and a_g_ZIF-62_P→T_ were measured by the IR microimaging technique to monitor the evolution of CO_2_ concentration over time. For this type of measurement, the focal plane array detector was employed, and the measurement window size was set to 50 µm × 150 µm. By placing the measurement window on an edge of the pressed glass, it was possible to observe how the CO_2_ molecules penetrate from the side as well as from the top and bottom surfaces. Since the measurement window was placed on the edge, the lowest part of the IR images is always blue, which corresponds to the external gas phase. Here the CO_2_ concentration is indicated by different colours: blue and red mean low and high CO_2_ concentration, respectively. The IR images were captured at different times from 0.35 min to 251.00 min for a_g_ZIF-62_P_ and from 0.25 min to 159.00 min for a_g_ZIF-62_P→T_.

### HR-TEM and ED measurements

All samples were ground in an agate mortar containing a few drops of EtOH. The resulting samples were diluted with EtOH and drop-cast onto 200-mesh copper grids, coated with a carbon film (Rigorous). Subsequently, the samples were dried in an oven at 70 °C. The HR-TEM and ED measurements were carried out at the Electron Microscopy Facility at IST Austria using the S/TEM JEOL JEM2800 instrument with an accelerating voltage of 200 kV equipped with a complementary metal–oxide–semiconductor transmission electron microscopy (TEM) camera (TemCam-XF416) for the HR-TEM images and a 1,024 × 1,024 pixel charge-coupled device camera (Hamamatsu ORCA R2) for the ED images. Although amorphous a_g_ZIF-62_nP_ and a_g_ZIF-62_P_ display a relatively robust beam tolerance, the crystalline ZIF-62 samples are very beam sensitive. To mitigate the effects of beam damage on ZIF-62, the electron beam was deliberately blocked on identifying the target crystals until the optimal magnification and focus distance were set. Subsequently, snapshots were taken to minimize any potential beam-induced alterations in the samples. The reported interatomic distance distributions were measured using the EM Measure Beta 0.85 software package. Reported values are averaged over a total of 200 counts, consisting of two independent counts of 100 counts per sample.

### Skeletal and envelope density measurements

Skeletal and envelope densities of the crystalline and glass samples were determined by He pycnometry (Ultrapyc, Anton Paar) and by Archimedes’ principle through immersion in toluene, respectively. To ensure that the same amount of material is measured in air as that in the liquid, glass vials were used to hold the sample during immersion. Sample-related buoyancy was derived through correction for the glass vial’s buoyancy, which, in turn, gives the sample density:

$${\rho }_{{\rm{s}}}=\frac{{m}_{{\rm{S,A}}}{\rho }_{{\rm{L}}}}{{m}_{{\rm{S,A}}}+{m}_{{\rm{C,A}}}-{m}_{{\rm{S+C,L}}}\,{-m}_{{\rm{C,A}}}\frac{{\rho }_{{\rm{L}}}}{{\rho }_{{\rm{C}}}}},$$

Here *ρ*_S_, *ρ*_L_ and *ρ*_C_ are the densities of the sample, liquid and container material, respectively. Equivalently, *m*_S,A_ and *m*_C,A_ are the individual masses of the sample and container as measured in air before immersion, respectively, whereas *m*_S+C,L_ is the mass determined for the sample and container combined when immersed in the liquid. Before measurement of the samples, the density of the glass vials used as the containers was determined with He pycnometry. The liquid’s density was derived immediately before sample measurement from the immersion of a reference material, namely, fused silica (Suprasil F300, Heraeus), with a well-known density of 2.2 g cm^−3^.

The observational errors for the densities determined via He pycnometry and Archimedes’ principle were estimated from error propagation with the errors of all the individual parameters and measurements.

In both methods, the reference fused silica material of approximately the same mass as the sample masses was measured in exactly the same way, which reproduced the expected value of 2.2 g cm^−3^ within the error range.

## Online content

Any methods, additional references, Nature Portfolio reporting summaries, source data, extended data, supplementary information, acknowledgements, peer review information; details of author contributions and competing interests; and statements of data and code availability are available at 10.1038/s41563-023-01738-3.

### Supplementary information

Supplementary Information Supplementary Figs. 1–29, Table 1, captions for Videos 1–5, captions for Extended Data Figs. 1 and 2, materials and text.

Supplementary Video 1 Explosion of the as-synthesized, not properly cleaned ZIF-62 crystal on melting in an Ar atmosphere. The decomposition of DMF and residuals leads to the explosion, as they act as blowing agents.

Supplementary Video 2 Melting a large, properly cleaned and dried ZIF-62 crystal in an inert Ar atmosphere. It is held at the melting point (*T*_m_ = 450 °C) for a long time. The pore collapse is visible through shrinking of the melt.

Supplementary Video 3 Melting smaller ZIF-62 crystals in an inert Ar atmosphere and keeping it at 480 °C above *T*_m_ to obtain a_g_ZIF-62_nP_ and visualize the surface energy of the liquid and better facilitate droplet formation.

Supplementary Video 4 Tempering of a_g_ZIF-62_nP_ at 400 °C (above *T*_g_ = 322 °C) in an inert Ar atmosphere to obtain a controlled pore collapse in a_g_ZIF-62_nP→T_. The shrinking is obvious.

Supplementary Video 5 Tempering of a shard of a_g_ZIF-62_P_ at 400 °C (above *T*_g_ = 322 °C) in an inert Ar atmosphere to obtain a controlled pore collapse in a_g_ZIF-62_P→T_. The shrinking is obvious.

### Source data

Source Data Fig. 1 Source data for Fig. 1d,f.

Source Data Fig. 2 Source data for Fig. 2b–f.

Source Data Fig. 3 Source data for Fig. 3e,f.

Source Data Fig. 4 Source data for Fig. 4d–f.

Source Data Fig. 5 Source data for Fig. 5b–d.

## Data Availability

All data supporting the findings in this study are available within this Article and its Supplementary Information. Source data are provided with this paper. Any additional data are available from the corresponding author upon request.

## References

[1] Sun X (2017). Metal-organic framework mediated cobalt/nitrogen-doped carbon hybrids as efficient and chemoselective catalysts for the hydrogenation of nitroarenes. ChemCatChem.

[2] Knebel A (2020). Solution processable metal-organic frameworks for mixed matrix membranes using porous liquids. Nat. Mater..

[3] Erdosy DP (2022). Microporous water with high gas solubilities. Nature.

[4] Bennett TD (2016). Melt-quenched glasses of metal-organic frameworks. J. Am. Chem. Soc..

[5] Bennett TD (2015). Hybrid glasses from strong and fragile metal-organic framework liquids. Nat. Commun..

[6] Xu W (2023). High-porosity metal-organic framework glasses. Angew. Chem. Int. Ed..

[7] Wondraczek L, Mauro JC (2009). Advancing glasses through fundamental research. J. Eur. Ceram. Soc..

[8] Li S (2019). Mechanical properties and processing techniques of bulk metal-organic framework glasses. J. Am. Chem. Soc..

[9] To T (2020). Fracture toughness of a metal-organic framework glass. Nat. Commun..

[10] Knebel A (2017). Defibrillation of soft porous metal-organic frameworks with electric fields. Science.

[11] Freund R (2021). 25 years of reticular chemistry. Angew. Chem. Int. Ed..

[12] Knebel A, Caro J (2022). Metal-organic frameworks and covalent organic frameworks as disruptive membrane materials for energy-efficient gas separation. Nat. Nanotechnol..

[13] Zhou S (2022). Asymmetric pore windows in MOF membranes for natural gas valorization. Nature.

[14] Thornton AW (2016). Porosity in metal-organic framework glasses. Chem. Commun..

[15] Zhou C (2018). Metal-organic framework glasses with permanent accessible porosity. Nat. Commun..

[16] Longley L (2019). Flux melting of metal-organic frameworks. Chem. Sci..

[17] Nozari, V. et al. Low‐temperature melting and glass formation of the zeolitic imidazolate frameworks ZIF‐62 and ZIF‐76 through ionic liquid incorporation. *Adv. Mater. Technol*. **7**, 2200343 (2022).

[18] Nozari V (2021). Ionic liquid facilitated melting of the metal-organic framework ZIF-8. Nat. Commun..

[19] Madsen RSK (2020). Ultrahigh-field ^67^Zn NMR reveals short-range disorder in zeolitic imidazolate framework glasses. Science.

[20] Qiao A (2018). A metal-organic framework with ultrahigh glass-forming ability. Sci. Adv..

[21] Frentzel-Beyme L, Kloß M, Kolodzeiski P, Pallach R, Henke S (2019). Meltable mixed-linker zeolitic imidazolate frameworks and their microporous glasses: from melting point engineering to selective hydrocarbon sorption. J. Am. Chem. Soc..

[22] Wang Y (2020). A MOF glass membrane for gas separation. Angew. Chem. Int. Ed..

[23] Yang Z (2023). ZIF-62 glass foam self-supported membranes to address CH_4_/N_2_ separations. Nat. Mater..

[24] Xia H (2022). A long-lasting TIF-4 MOF glass membrane for selective CO_2_ separation. J. Mem. Sci..

[25] Nozari V, Calahoo C, Longley L, Bennett TD, Wondraczek L (2020). Structural integrity, meltability, and variability of thermal properties in the mixed-linker zeolitic imidazolate framework ZIF-62. J. Chem. Phys..

[26] Healy C (2020). The thermal stability of metal-organic frameworks. Coord. Chem. Rev..

[27] Madsen RSK (2022). Mixed metal node effect in zeolitic imidazolate frameworks. RSC Adv..

[28] Stepniewska M, Østergaard MB, Zhou C, Yue Y (2020). Towards large-size bulk ZIF-62 glasses via optimizing the melting conditions. J. Non Cryst. Solids.

[29] Frentzel-Beyme L (2019). Porous purple glass—a cobalt imidazolate glass with accessible porosity from a meltable cobalt imidazolate framework. J. Mater. Chem. A.

[30] Berens S (2018). Ethane diffusion in mixed linker zeolitic imidazolate framework-7-8 by pulsed field gradient NMR in combination with single crystal IR microscopy. Phys. Chem. Chem. Phys..

[31] Chmelik C (2011). Nanoporous glass as a model system for a consistency check of the different techniques of diffusion measurement. Chem. Phys. Chem..

[32] Titze T (2015). Transport in nanoporous materials including MOFs: the applicability of Fick’s laws. Angew. Chem. Int. Ed..

[33] Gustafsson M, Zou X (2013). Crystal formation and size control of zeolitic imidazolate frameworks with mixed imidazolate linkers. J. Porous Mater..

[34] Banerjee R (2008). High-throughput synthesis of zeolitic imidazolate frameworks and application to CO_2_ capture. Science.

[35] Widmer RN (2019). Pressure promoted low-temperature melting of metal-organic frameworks. Nat. Mater..

[36] Gandara-Loe J, Bueno-Perez R, Missyul A, Fairen-Jimenez D, Silvestre-Albero J (2021). Molecular sieving properties of nanoporous mixed-linker ZIF-62: associated structural changes upon gas adsorption application. ACS Appl. Nano Mater..

[37] Frentzel-Beyme L, Kolodzeiski P, Weiß J-B, Schneemann A, Henke S (2022). Quantification of gas-accessible microporosity in metal-organic framework glasses. Nat. Commun..

[38] Shi Z, Arramel A, Bennett TD, Yue Y, Li N (2022). The deformation of short-range order leading to rearrangement of topological network structure in zeolitic imidazolate framework glasses. iScience.

[39] Kärger J (2014). Microimaging of transient guest profiles to monitor mass transfer in nanoporous materials. Nat. Mater..

[40] Yang Q (2020). Capillary condensation under atomic-scale confinement. Nature.

[41] Breunig HM, Rosner F, Lim T-H, Peng P (2023). Emerging concepts in intermediate carbon dioxide emplacement to support carbon dioxide removal. Energy Environ. Sci..

[42] Morris W, Doonan CJ, Furukawa H, Banerjee R, Yaghi OM (2008). Crystals as molecules: postsynthesis covalent functionalization of zeolitic imidazolate frameworks. J. Am. Chem. Soc..

