# Supplementary material for: Precise control over gas-transporting channels in zeolitic imidazolate framework glasses
Source: Nat Mater. 2023 Dec 20;23(2):262–70. doi: 10.1038/s41563-023-01738-3 (PMC10837076; doi:10.1038/s41563-023-01738-3)
Supplement: Supplementary file 1 — Supplementary Figs. 1–29, Table 1, captions for Videos 1–5, captions for Extended Data Figs. 1 and 2, materials and text. [file 41563_2023_1738_MOESM1_ESM.pdf]

nature materials

Article

https://doi.org/10.1038/s41563-023-01738-3

Precise control over gas-transporting channels in zeolitic imidazolate framework glasses

In the format provided by the authors and unedited

This PDF file includes:

- Materials
- Supplementary Text
- Figs. S1 to S29
- Table S1
- Captions for Videos S1 to S5
- Captions for Extended Data figures S1 to S2

Other Supplementary Information for this manuscript include the following:

- Videos S1 to S5
- Extended data figures 1 to 2

Materials

Zinc nitrate hexahydrate (≥99%) and cobalt (II) nitrate hexahydrate (≥99%) were purchased from ABCR. Benzimidazole (≥99%) was supplied by Alfa Aesar and Imidazole (≥99.5%) was purchased from Sigma-Aldrich. N,N-Dimethylformamide (≥99.9%) was supplied by VWR, dichloromethane (≥99%, stabilized with ethanol) and toluene (≥99.8%) – by Acros Organics.

Supplementary Text

Volume loss calculations

For the series of a_gZIF-62_P samples its volume loss while tempering (a_gZIF-$62_{P\rightarrow T}$) was calculated geometrically. Approximate area of the surface was multiplied by the sample's thickness. Parameters for these calculations were obtained from a built-in software of the microscope (**Fig. S12** to **S16**). The thickness of each sample before and after tempering was measured separately. The errors are non-sampling and were assumed based on the numbers of surface defects for each sample individually (**Table S1**).

Diffusion coefficients calculation

The obtained uptake curves in diffusion measurements were fitted with the following solutions of Fick's second law of diffusion, assuming that the uptake is solely diffusion-limited, in order to calculate the diffusion coefficients D:

For spherical sample,

$$\frac{m(t)}{m(\infty)} = 1 - \frac{6}{\pi^2}\sum_{n=1}^{\infty}\frac{1}{n^2}exp\left(\frac{-Dn^2\pi^2t}{r^2}\right)$$

where $m(t)$ and $m(\infty)$ are amounts of guest molecules at time t and in equilibrium, respectively. D is diffusion coefficient and r is radius of the spherical sample.

For 1D plane-parallel sample,

$$\frac{m(t)}{m(\infty)} = 1 - \frac{8}{\pi^2}\sum_{n=0}^{\infty}\frac{1}{(2n+1)^2}exp\left(\frac{-D(2n+1)^2\pi^2t}{L^2}\right)$$

where L thickness of the sample.

For the sake of simplicity, ZIF-62(Zn), a_gZIF-62_{nP} and a_gZIF-$62_{nP\rightarrow T}$ were assumed to be spherical. On the other hand, a_gZIF-62_P and a_gZIF-$62_{P\rightarrow T}$ were assumed to be 1D plane-parallel since the diffusion measurements were measured at the center of the pressed glass pieces and, considering relatively slow diffusion of guest molecules in those glasses, the influence of the molecules diffusing from the four sides would be negligible. The calculated diffusion coefficients are shown in **Table 1** in the main text.

Fig. S1.

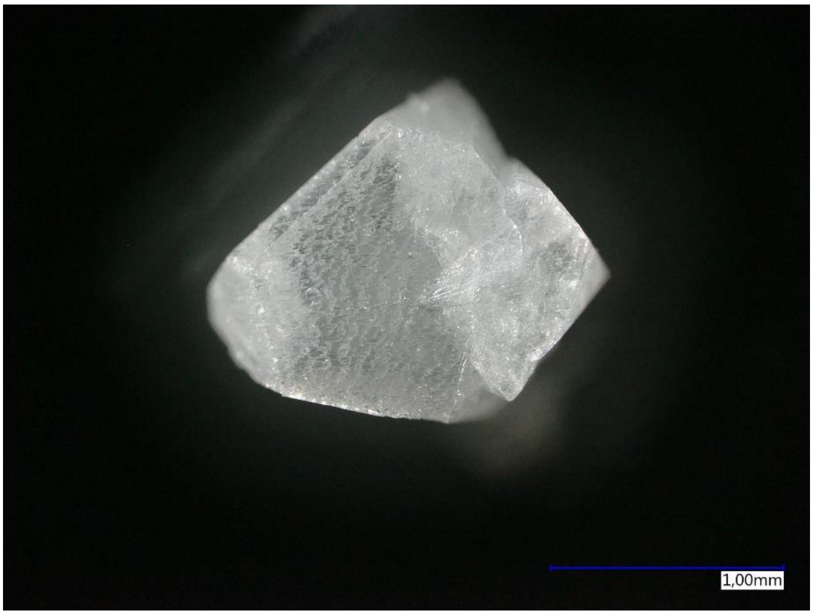

Microscope photograph of a large ZIF-62(Zn) crystal.

Fig. S2.

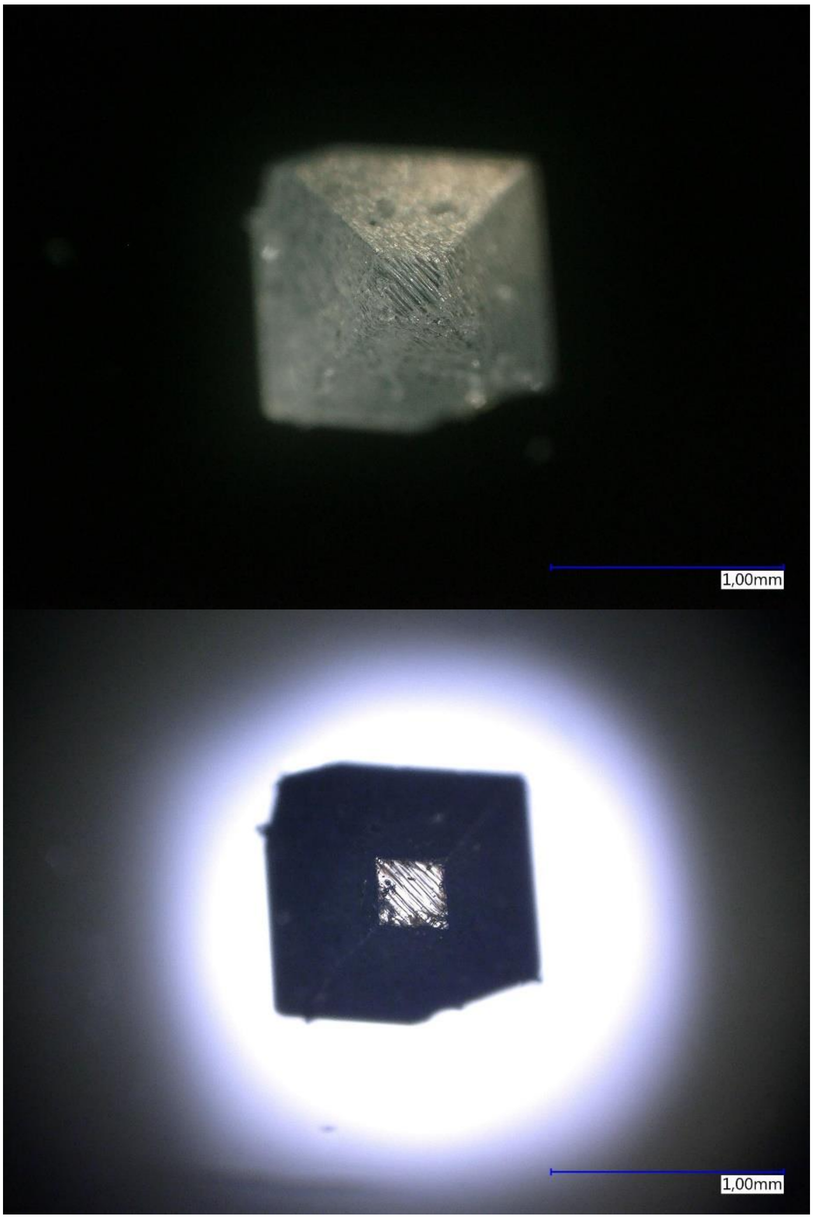

Microscope photographs of ZIF-62(Zn) crystal with different light modes demonstrating its transparency.

Fig. S3.

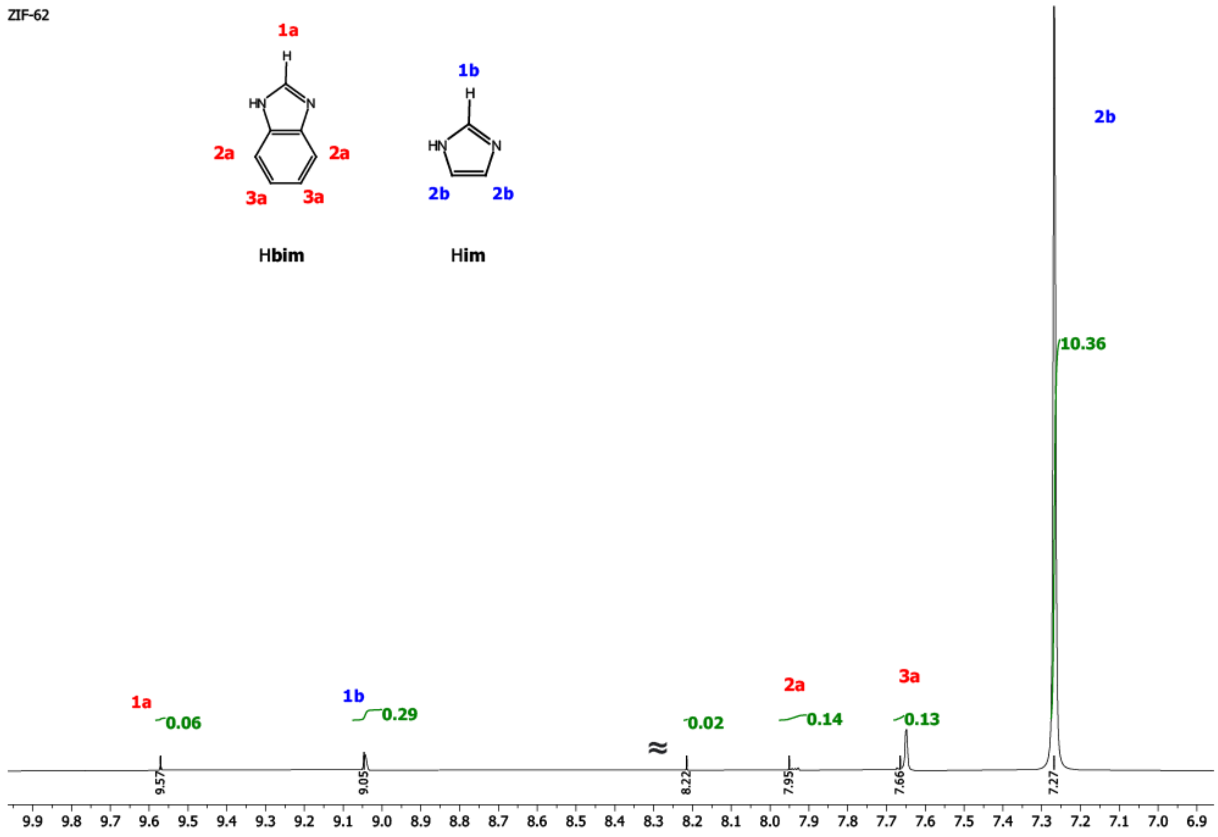

^{1}H NMR spectra of ZIF-62(Zn). δH (500 MHz; DCl (20%)/D_2O (0.889 mL) and DMSO-d_6 (3 mL); Me_4Si), 9.57 (1H, s, H_b), 9.05 (1H, s, H_a), 2.67 (DMSO), 0.00 (TMS).

Fig. S4.

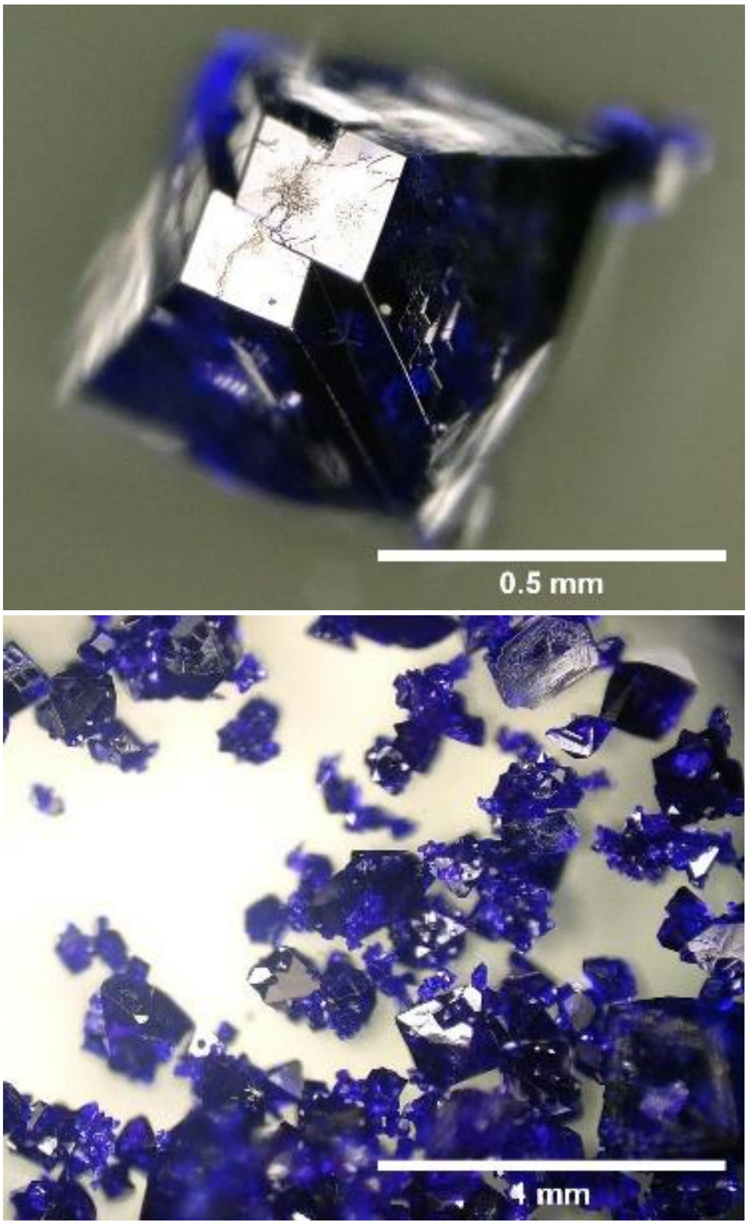

Microscope photographs of crystalline ZIF-62(Co).

Fig. S5.

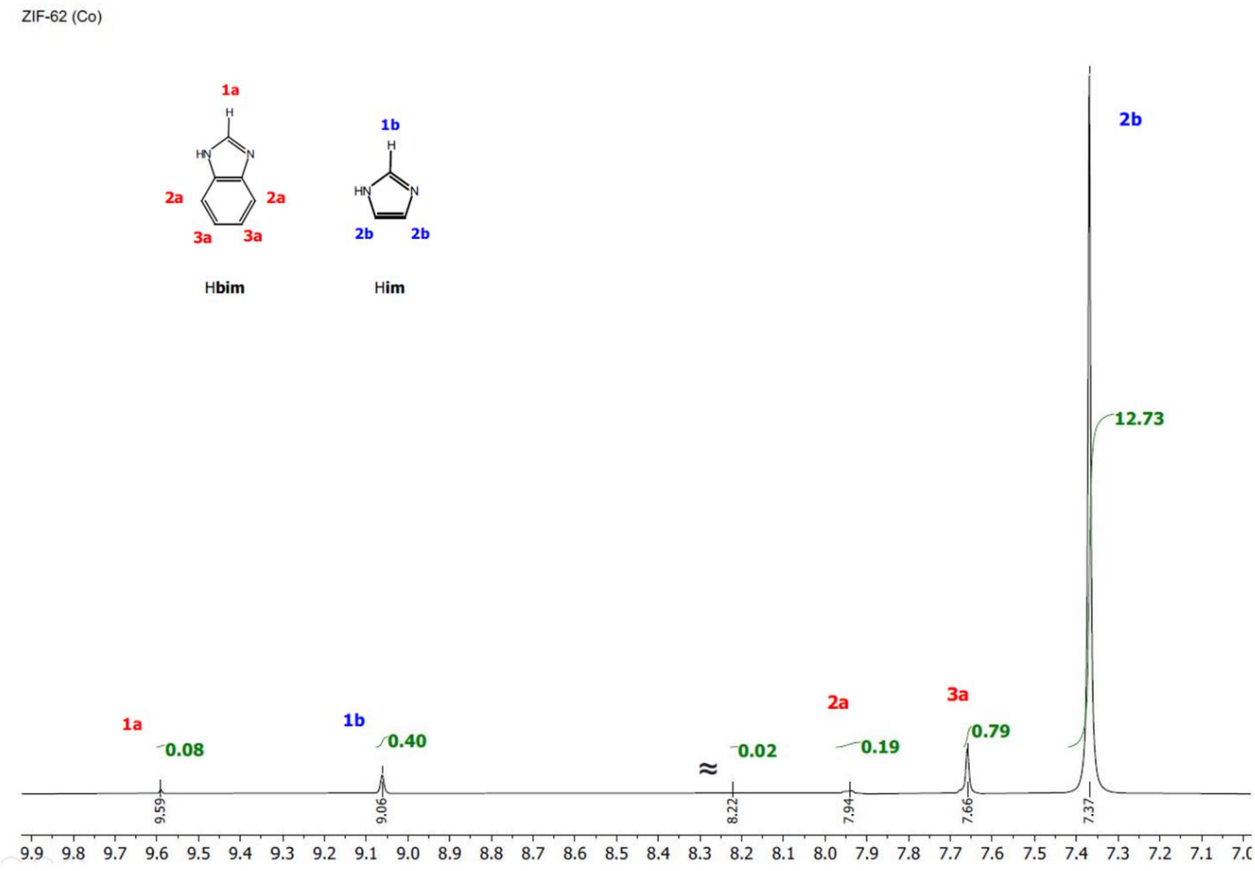

^{1}H NMR spectra of ZIF-62(Co). δH (500 MHz; DCl (20%)/D_2O (0.889 mL) and DMSO-d_6 (3 mL); Me_4Si), 9.58 (1H, s, H_b), 9.05 (1H, s, H_a), 2.67 (DMSO), 0.00 (TMS).

Fig. S6.

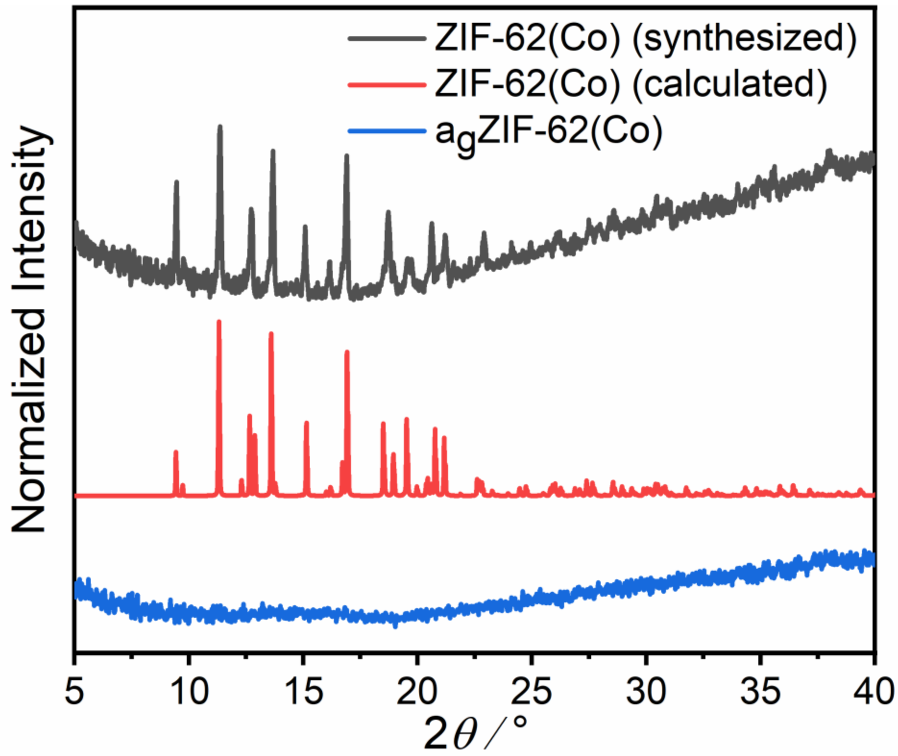

PXRD of the phase-pure ZIF-62(Co) and a_gZIF-62(Co) with corresponding calculated pattern. The background appearance is caused by the fluorescence background correction for the cobalt containing samples.

Fig. S7.

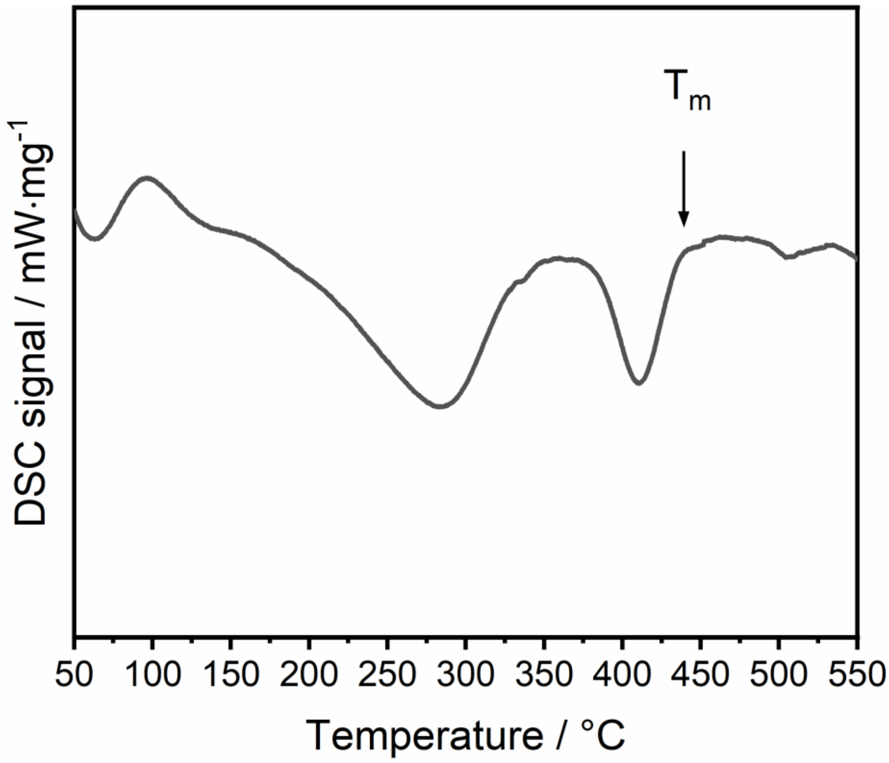

DSC measurement for ZIF-62(Co).

Fig. S8.

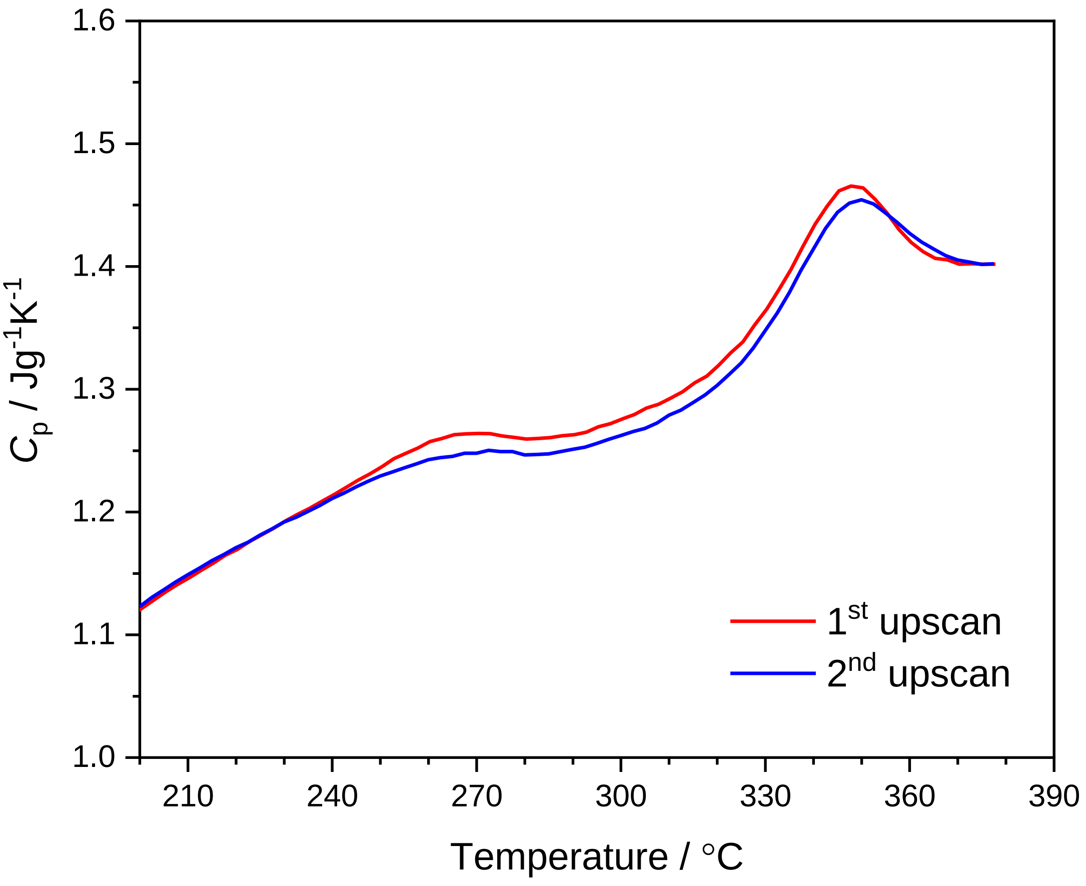

Cyclic C_p scan of ZIF-62(Co).

Fig. S9.

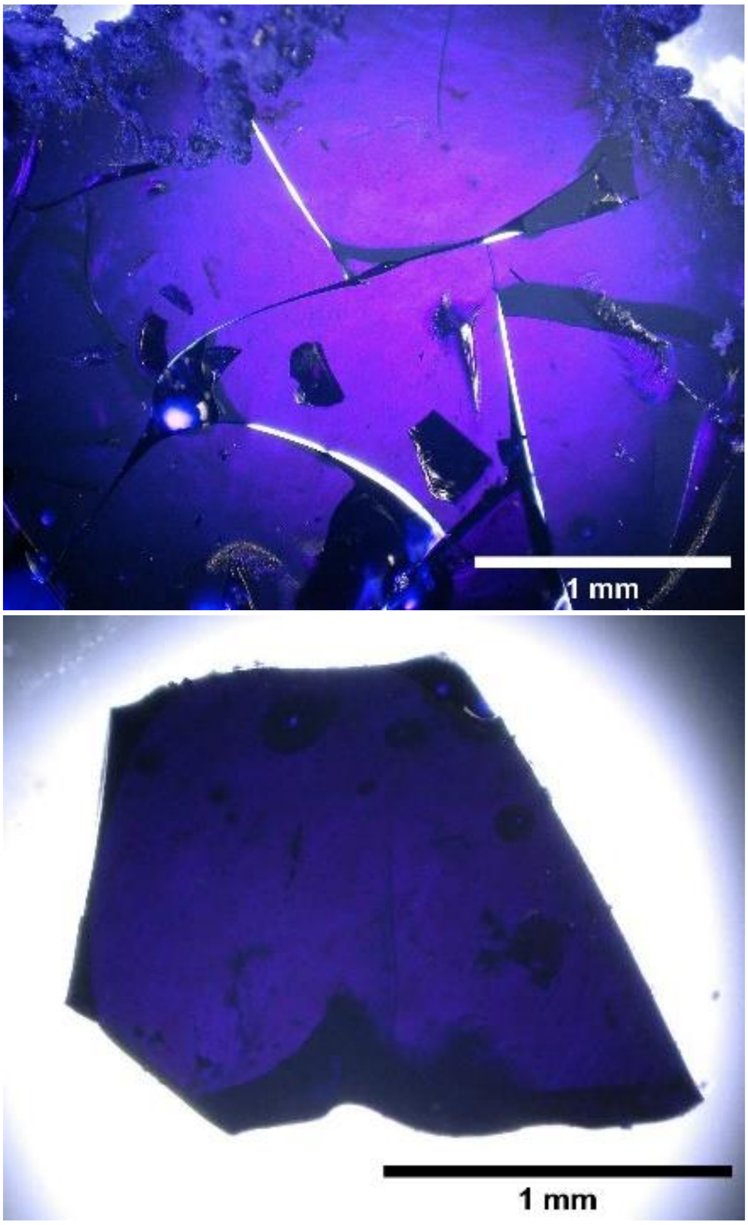

Microscope photographs of ZIF-62(Co) pressed glass.

Fig. S10.

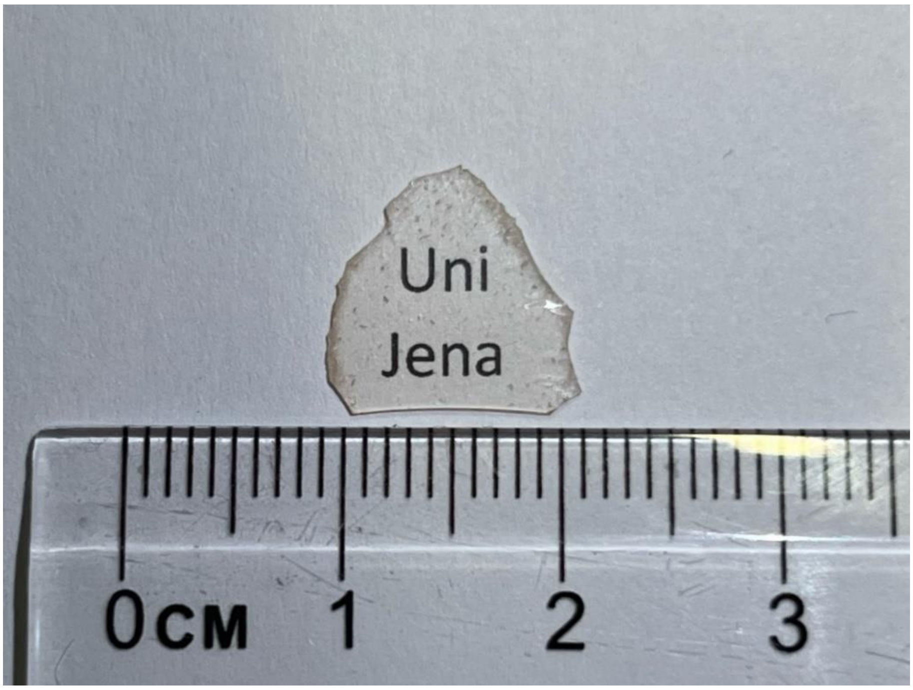

Demonstration of a larger $a_gZIF\text{-}62_P(Zn)$ piece.

Fig. S11.

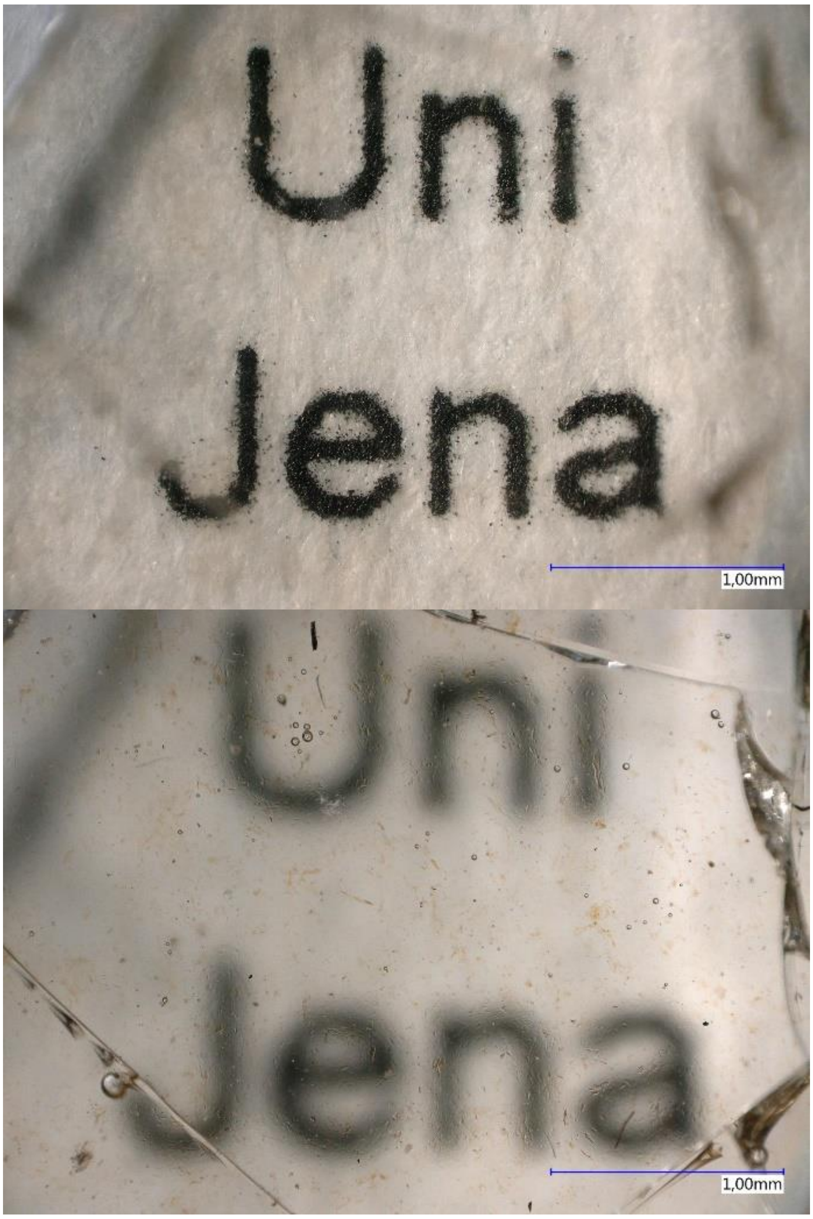

Demonstration of a transparency of $a_gZIF\text{-}62_P(Zn)$: microscope photographs of the clearly visible through the sample inscription with a focus on the inscription (top) and glass (bottom).

Fig. S12.

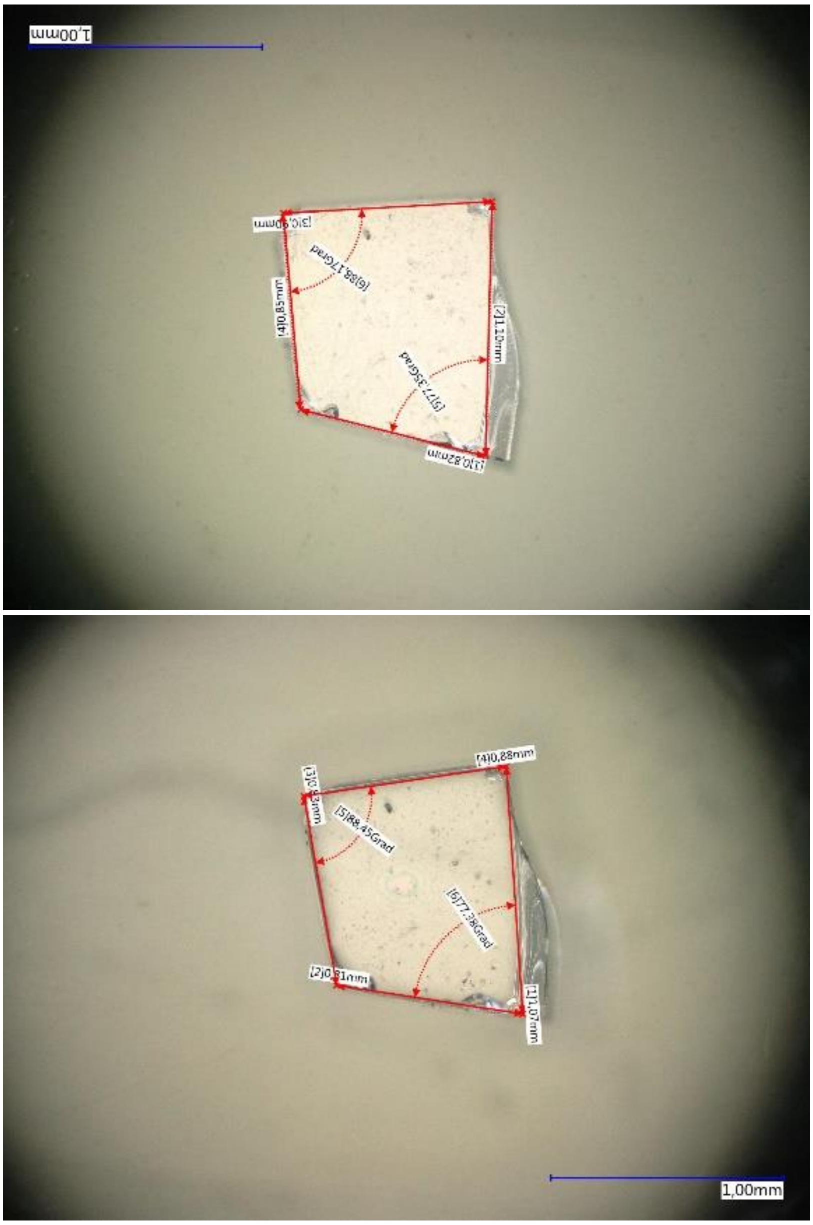

a_gZIF-62$_P$ (top) and a_gZIF-62$_{P\rightarrow T}$ (bottom) with tempering time of 10 minutes and the geometrical parameters required for the area determination, obtained from the built-in microscope software.

Fig. S13.

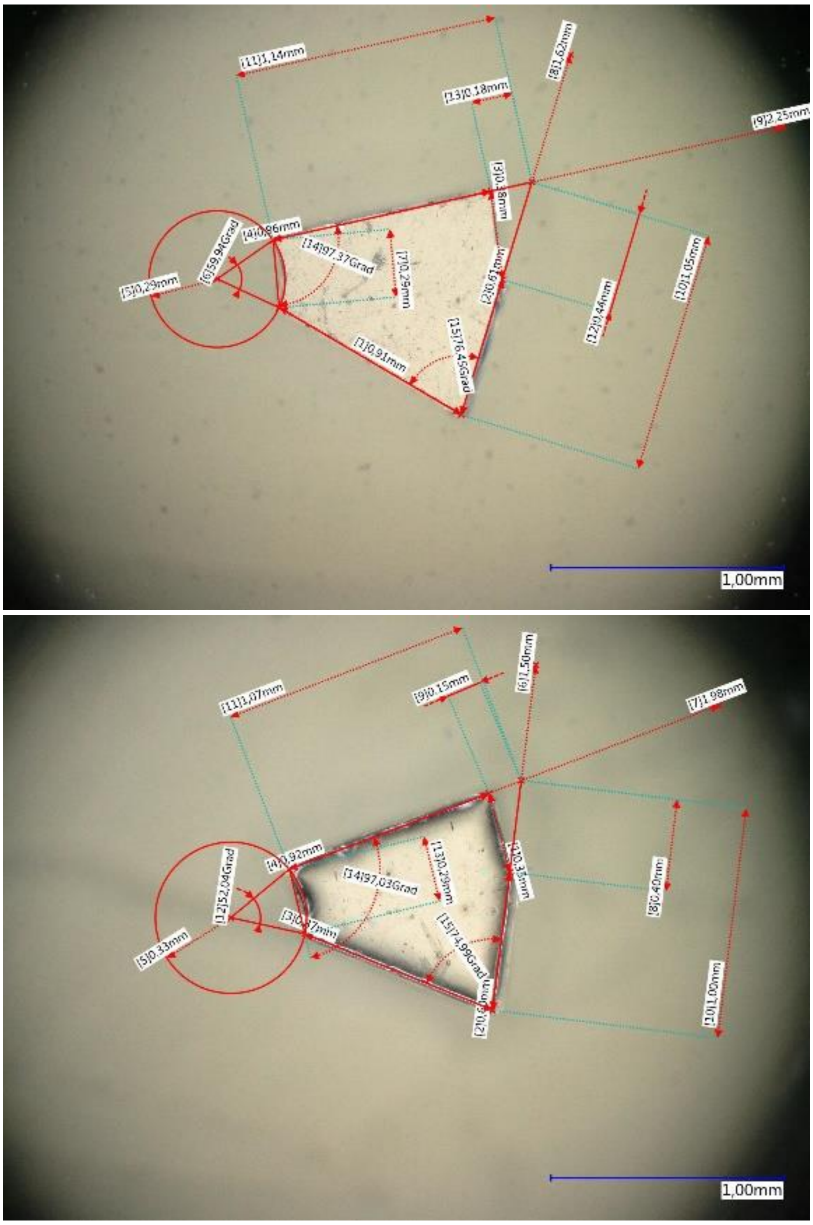

$a_gZIF\text{-}62_P$ (top) and $a_gZIF\text{-}62_{P\rightarrow T}$ (bottom) with tempering time of 30 minutes and the geometrical parameters required for the area determination, obtained from the built-in microscope software.

Fig. S14.

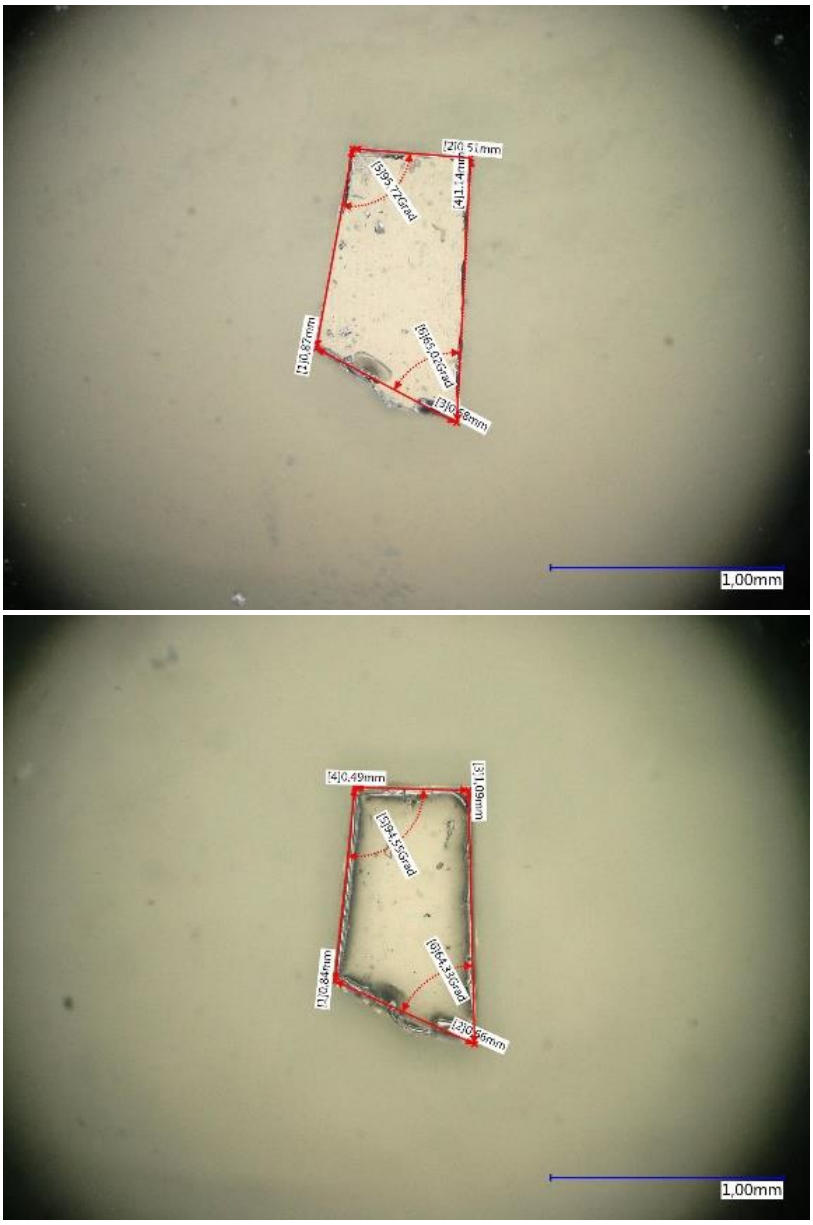

$_a$ $_g$ZIF-62$_P$ (top) and $_a$ $_g$ZIF-62$_{P\rightarrow T}$ (bottom) with tempering time of 60 minutes and the geometrical parameters required for the area determination, obtained from the built-in microscope software.

Fig. S15.

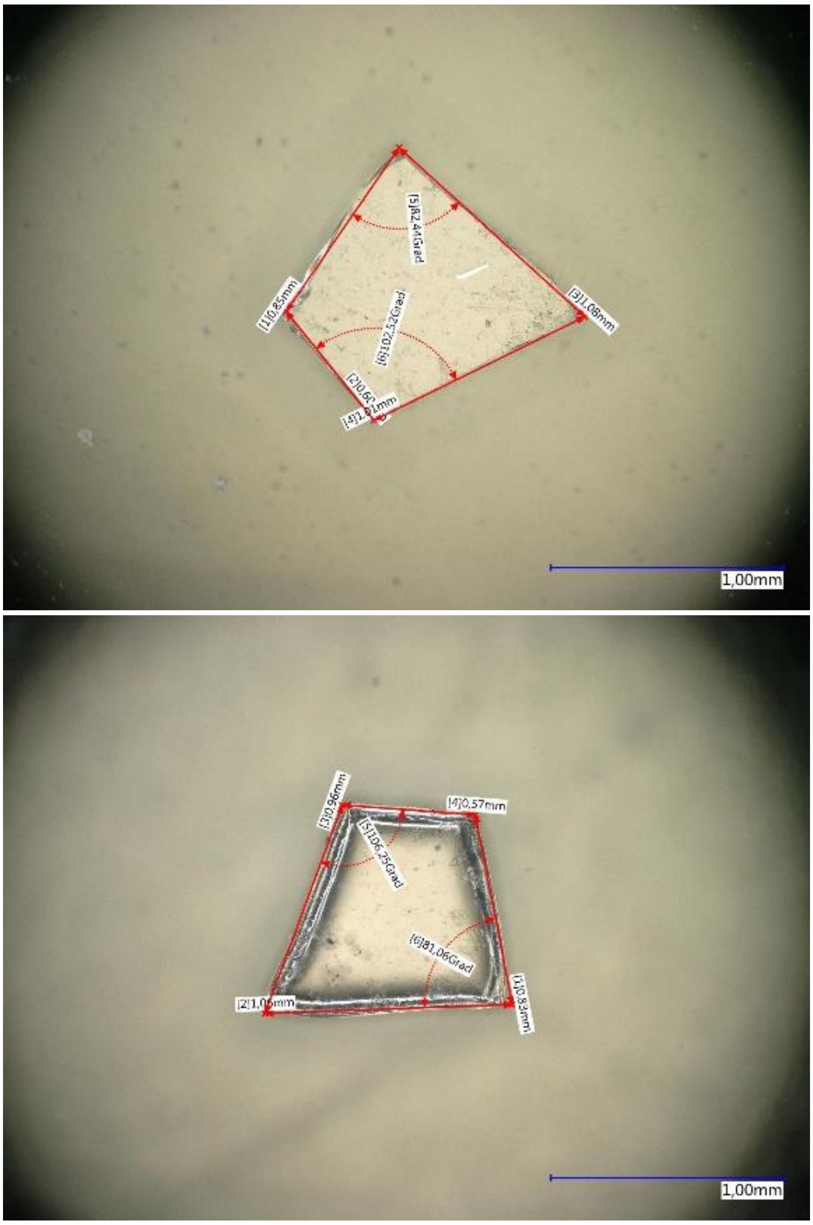

a_gZIF-62$_P$ (top) and a_gZIF-62$_{P\rightarrow T}$ (bottom) with tempering time of 120 minutes and the geometrical parameters required for the area determination, obtained from the built-in microscope software.

Fig. S16.

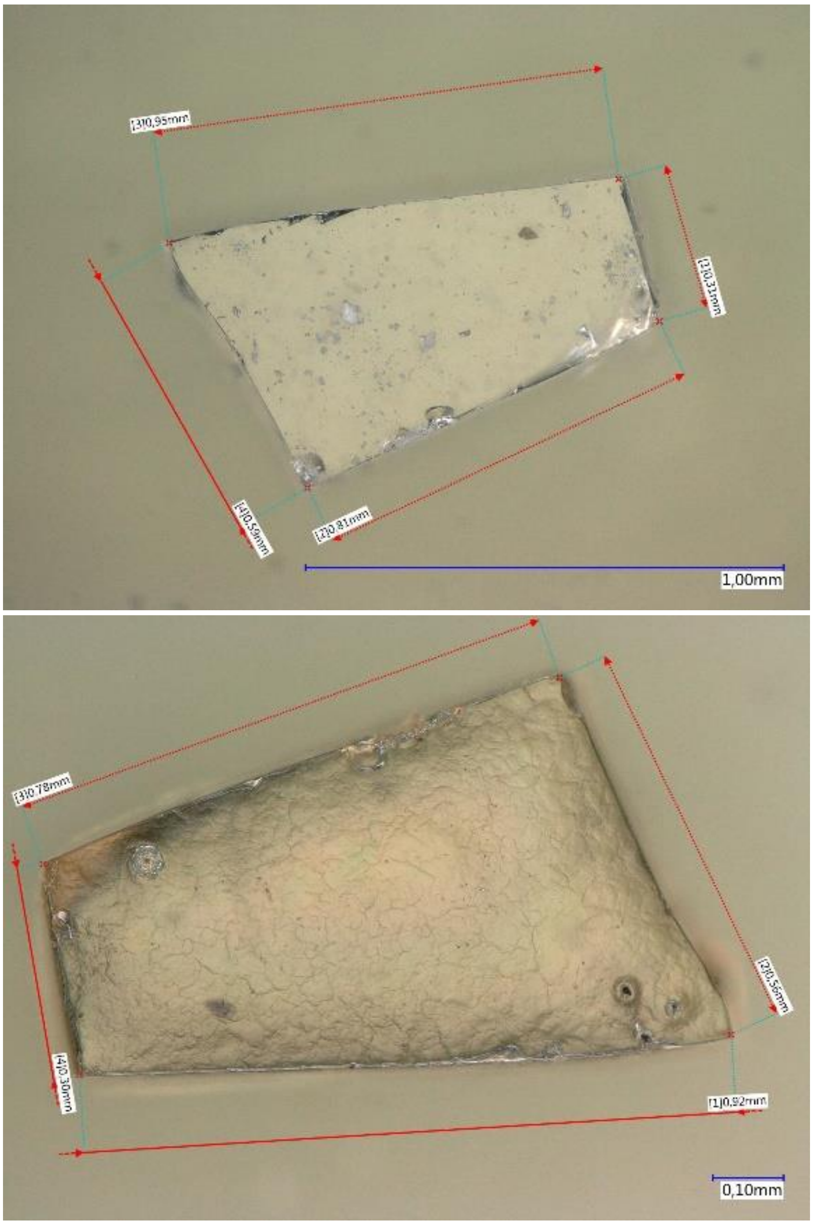

a_gZIF-62$_P$ (top) and a_gZIF-62$_{P\rightarrow T}$ (bottom) with tempering time of 240 minutes and the geometrical parameters required for the area determination, obtained from the built-in microscope software.

Fig. S17.

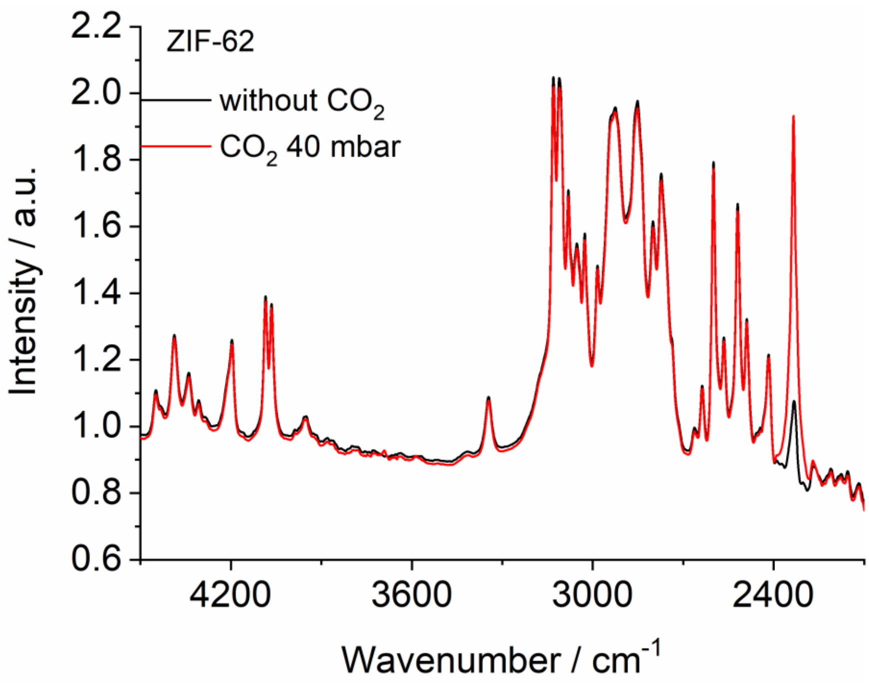

IR spectrum of ZIF-62 with and without CO_2 in the cell.

Fig. S18.

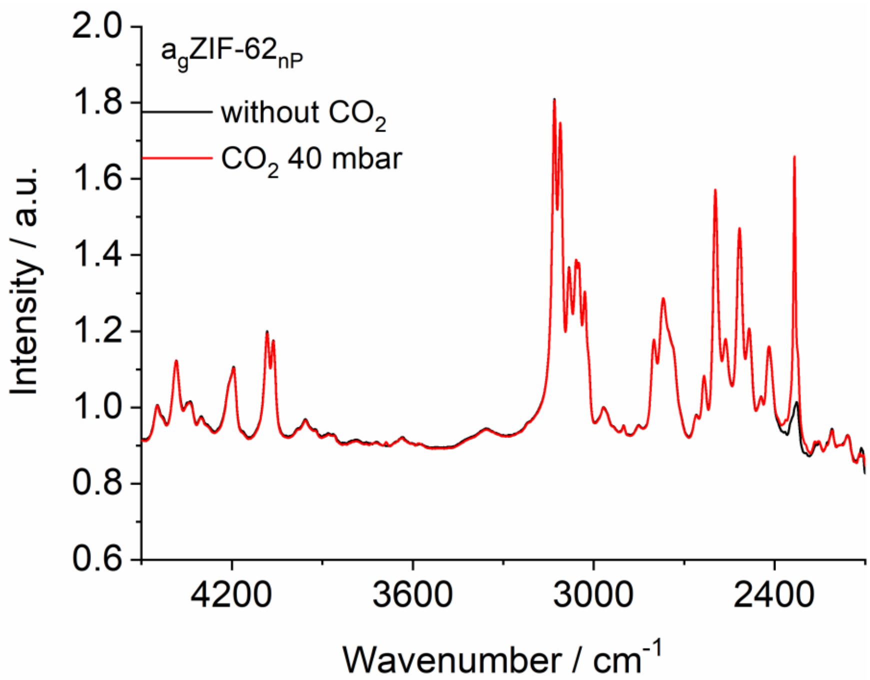

IR spectrum of $a_gZIF\text{-}62_{nP}$ with and without CO_2 in the cell.

Fig. S19.

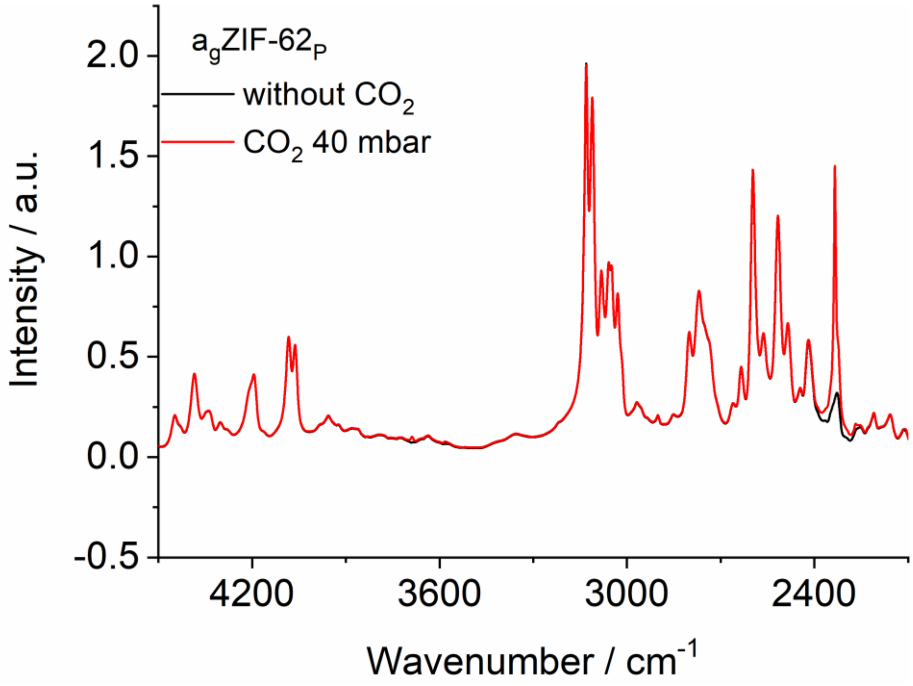

IR spectrum of $a_gZIF\text{-}62_P$ with and without CO_2 in the cell.

Fig. S20.

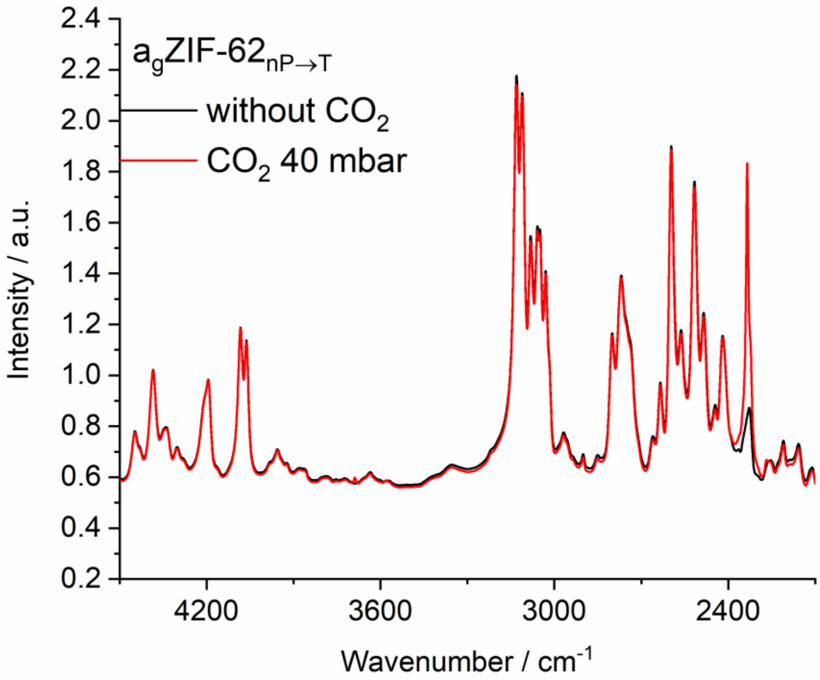

IR spectrum of a_gZIF-62$_{nP\rightarrow T}$ with and without CO_2 in the cell.

Fig. S21.

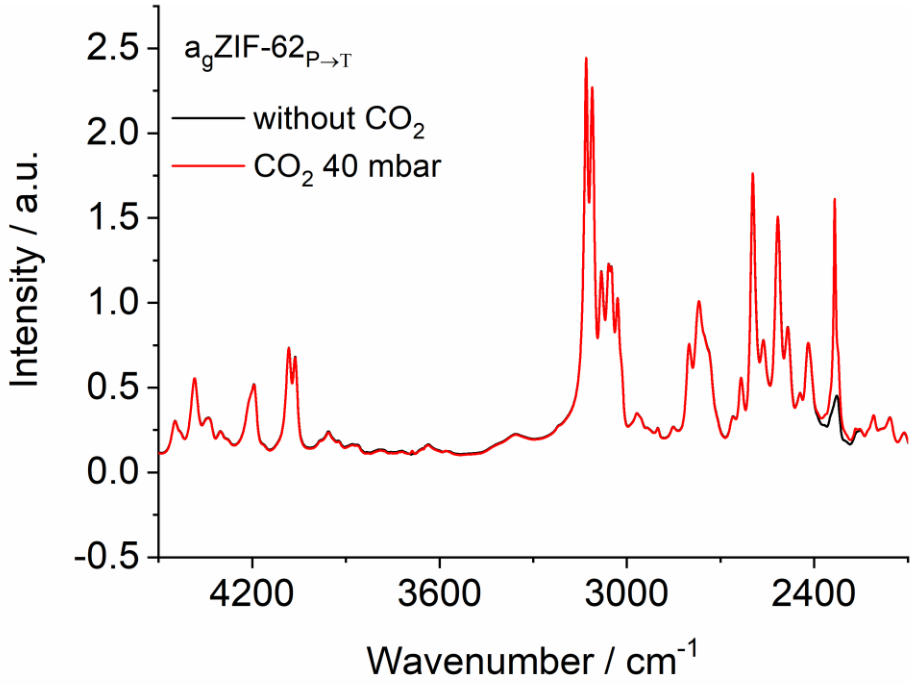

IR spectrum of $a_gZIF-62_{P\rightarrow T}$ with and without CO_2 in the cell.

Fig. S22.

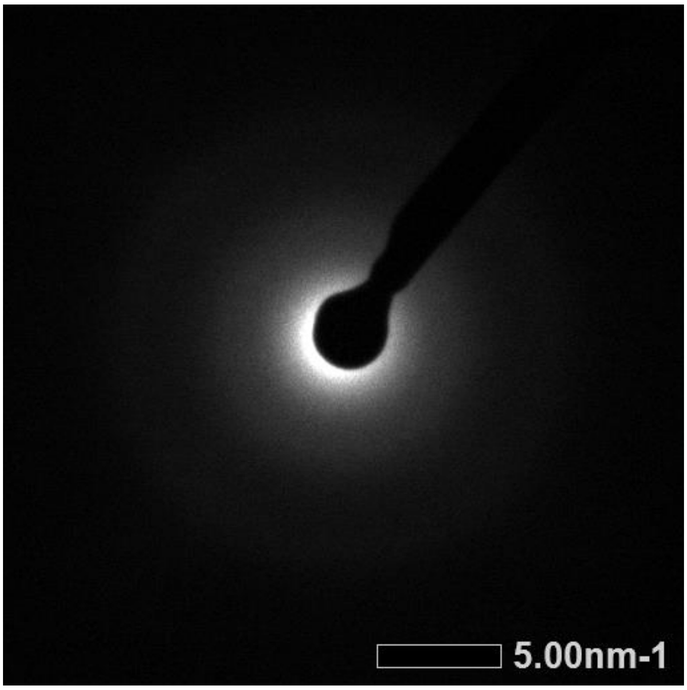

Halo-like electron diffraction pattern of amorphous $a_gZIF\text{-}62_{nP}$.

Fig. S23.

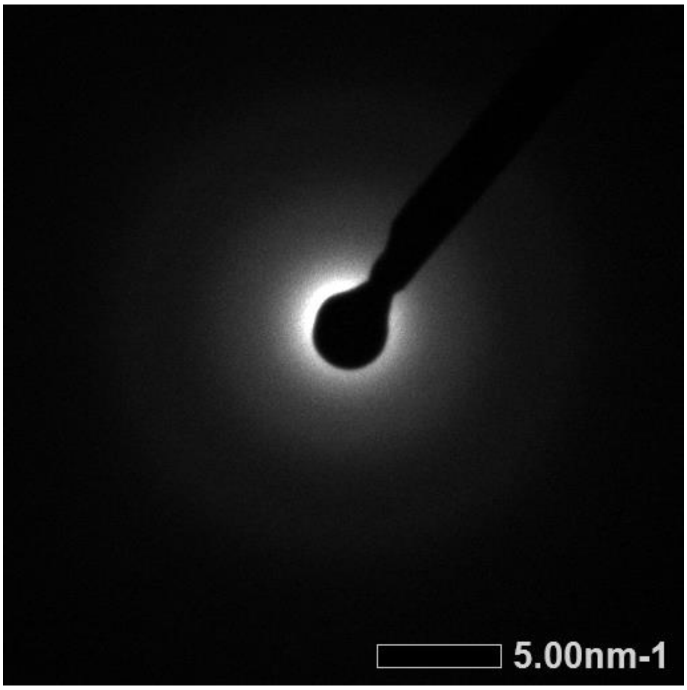

Halo-like electron diffraction pattern of amorphous $a_gZIF\text{-}62_P$.

Fig. S24.

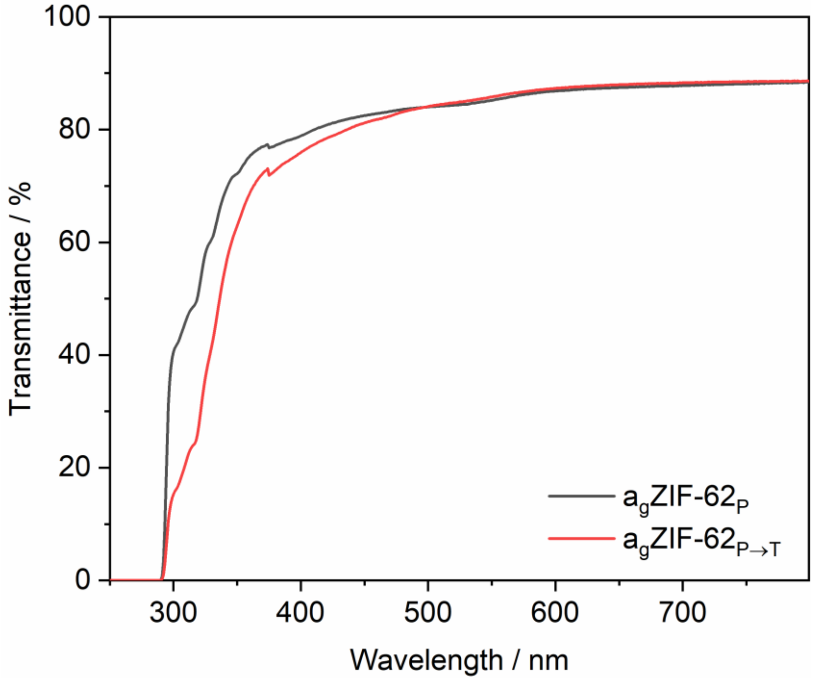

UV-vis transmittance spectra of $a_gZIF\text{-}62_P$ and $a_gZIF\text{-}62_{P\rightarrow T}$, tempered for 4 hours.

Fig. S25.

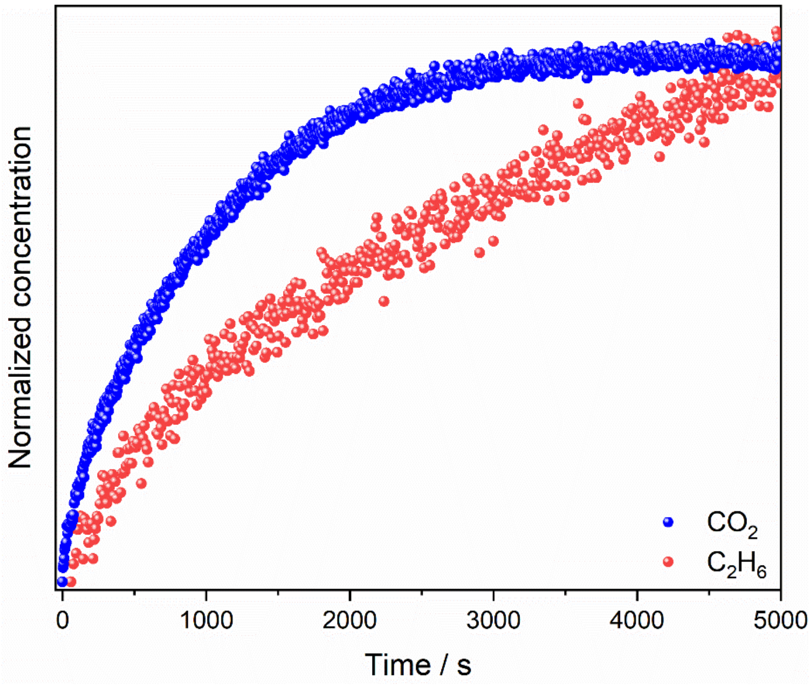

Normalized kinetic gas uptake curves for 0-40 mbar CO_2 and 0-200 mbar ethane in a_gZIF-$62_{nP \rightarrow T}$, tempered in the inert Ar atmosphere.

Fig. S26.

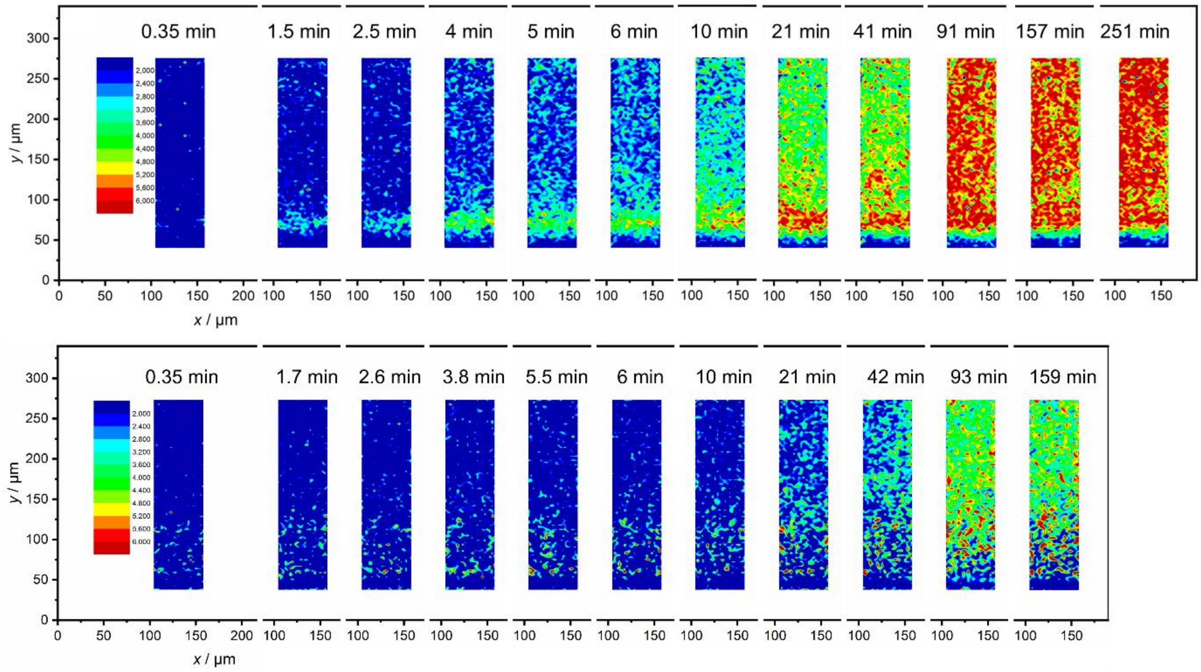

A comparison of guest concentration mapping upon CO_2 uptake in a_gZIF-62$_P$ (top) and a_gZIF-62$_{P\rightarrow T}$ (bottom) on the edge of the glass shards (blue color means relatively low CO_2 concentration and the gradient to red color with high CO_2 concentration).

Fig. S27.

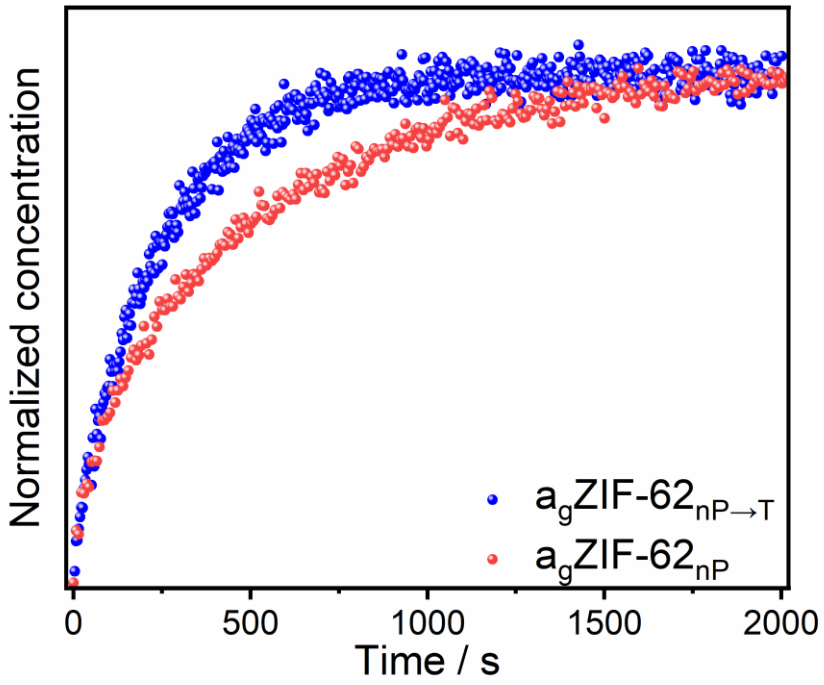

Normalized kinetic gas uptake curves for 0-40 mbar CO_2 in a_gZIF-62$_{nP}$ and a_gZIF-62$_{nP\rightarrow T}$, tempered in the air atmosphere.

Fig. S28.

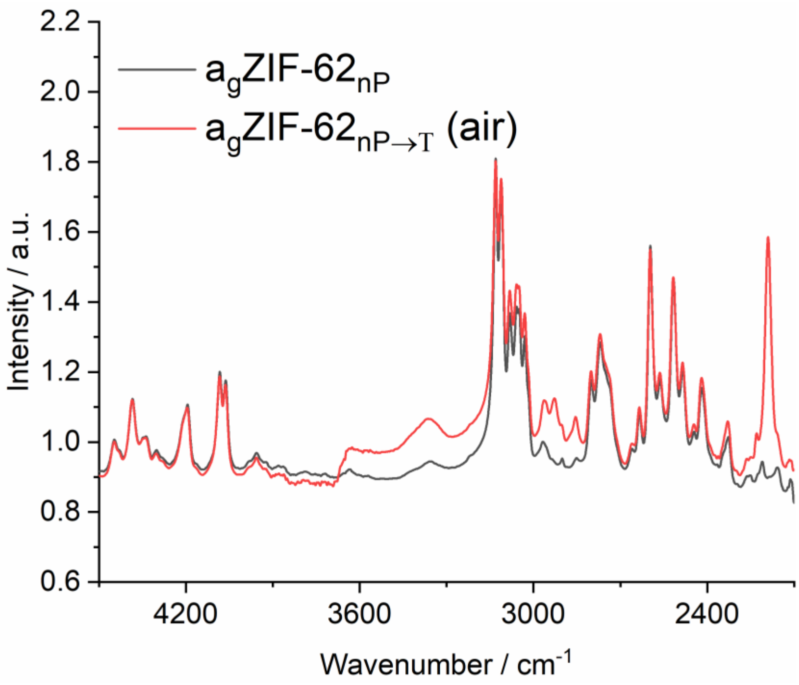

IR spectrum of $a_gZIF\text{-}62_{nP}$ and $a_gZIF\text{-}62_{nP\rightarrow T}$, tempered in air.

Fig. S29.

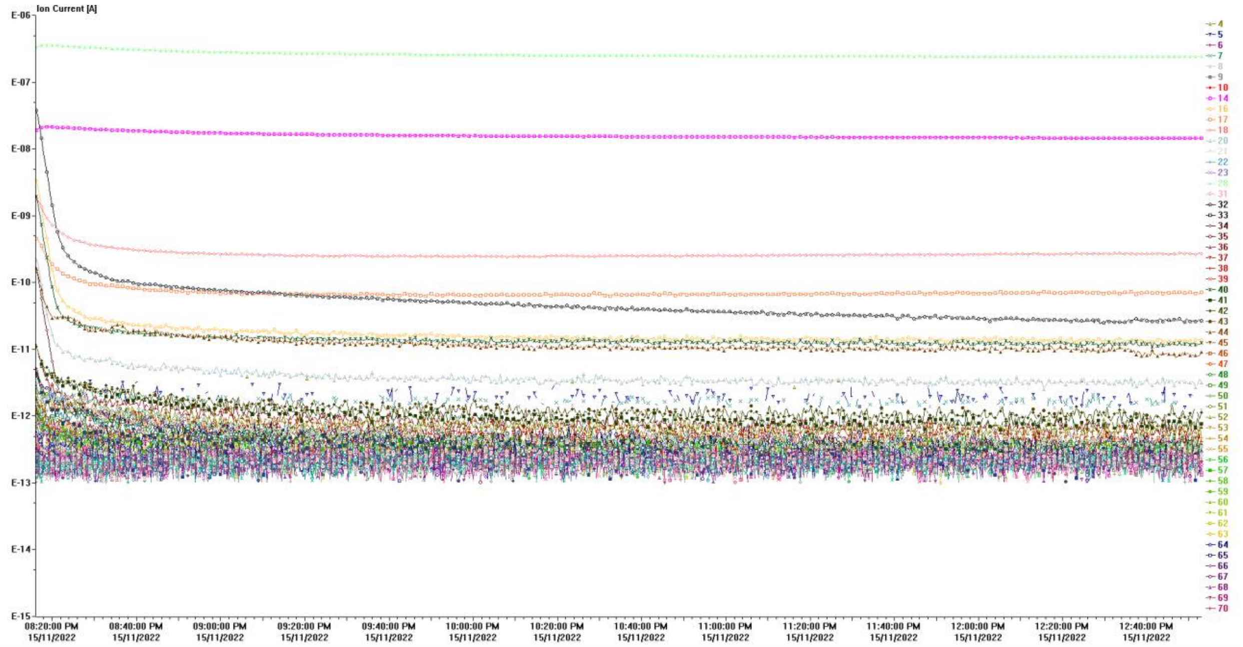

Mass spectrometry data for $a_gZIF\text{-}62_{nP\rightarrow T}$ tempering.

Table S1.

	Before tempering					After tempering				
Tempering (min)	10	30	60	120	240	10	30	60	120	240
Area (mm^2)	0,822	0,586	0,572	0,751	0,385	0,788	0,541	0,530	0,698	0,354
Thickness (mm)	0,21	0,21	0,24	0,22	0,21	0,20	0,20	0,22	0,2	0,19
Volume (mm^3)	0,172	0,123	0,137	0,165	0,081	0,158	0,108	0,117	0,140	0,067
Volume loss (%)						8,25 ±0,58	12,08 ±0,48	15,06 ±1,96	15,51 ±0,93	16,81 ±1,18

Geometrical parameters and volume loss calculated for $a_gZIF\text{-}62_{P\rightarrow T}$ samples.

Video S1.

Exploding of the as-synthesized, not properly cleaned ZIF-62 crystal upon melting in Ar atmosphere. The decomposition of DMF and residuals leads to the explosion, as they act as blowing agents.

Video S2.

Melting a large, properly cleaned and dried ZIF-62 crystal in the inert Ar atmosphere. It is held at the melting point (T_m = 450 °C) for a long time. The pore collapse is visible through shrinking of the melt.

Video S3.

Melting smaller ZIF-62 crystals in the inert Ar atmosphere and keeping it at 480 °C above T_m to obtain a_gZIF-62_{nP} and visualize the surface energy of the liquid and further to droplet formation better.

Video S4.

Tempering of a_gZIF-62_{nP} at 400°C (above T_g = 322°C) in the inert Ar atmosphere to obtain a controlled pore-collapse in a_gZIF-$62_{nP\rightarrow T}$. The shrinking is obvious.

Video S5.

Tempering of a shard of a_gZIF-62_{P} at 400°C (above T_g = 322 °C) in the inert Ar atmosphere to obtain a controlled pore-collapse in a_gZIF-$62_{P\rightarrow T}$. The shrinking is obvious.

Extended data figure 1.

The statistical values of the interatomic distances in $a_gZIF\text{-}62_{nP}$ obtained through HR-TEM with blue lines corresponding to the distance measured.

Extended data figure 2.

The statistical values of the interatomic distances in $a_gZIF\text{-}62_{P}$ obtained through HR-TEM with blue lines corresponding to the distance measured.
